# Probing Protein–Ligand Methyl−π Interaction Geometries through Chemical Shift Measurements of Selectively Labeled Methyl Groups

**DOI:** 10.1021/acs.jmedchem.4c01128

**Published:** 2024-07-29

**Authors:** Andreas Beier, Gerald Platzer, Theresa Höfurthner, Aleksandra L. Ptaszek, Roman J. Lichtenecker, Leonhard Geist, Julian E. Fuchs, Darryl B. McConnell, Moriz Mayer, Robert Konrat

**Affiliations:** † Christian Doppler Laboratory for High-Content Structural Biology and Biotechnology, Department of Structural and Computational Biology, Max Perutz Laboratories, University of Vienna, Campus Vienna Biocenter 5, 1030 Vienna, Austria; ‡ Christian Doppler Laboratory for High-Content Structural Biology and Biotechnology, Institute of Organic Chemistry, University of Vienna, Währingerstraße 38, 1090 Vienna, Austria; § Vienna Doctoral School of Chemistry, University of Vienna, Währingerstraße 38, 1090 Vienna, Austria; ∥ Department of Structural and Computational Biology, Max Perutz Laboratories, Campus Vienna Biocenter 5, 1030 Vienna, Austria; ⊥ Boehringer Ingelheim RCV GmbH & Co. KG, Dr. Boehringergasse 5-11, 1121 Vienna, Austria; # MAG-LAB, Karl-Farkas-Gasse 22, 1030 Vienna, Austria; ∇ Curie Bio, 177 Huntington Ave Ste 1703, Boston, Massachusetts 02115-3153, United States

## Abstract

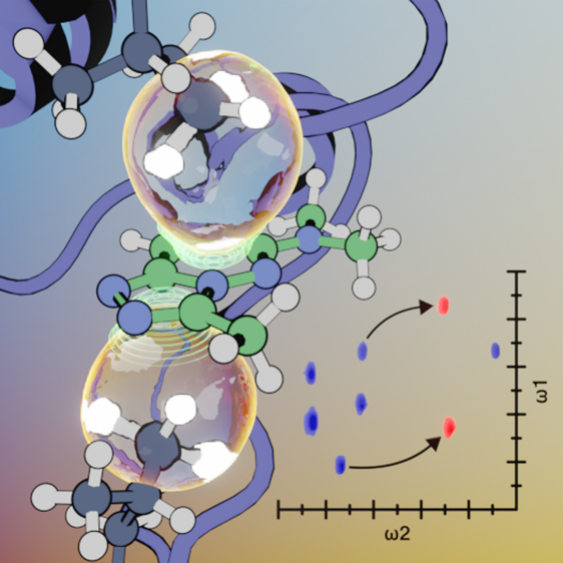

Fragment-based drug design is heavily dependent on the optimization of initial low-affinity binders. Herein we introduce an approach that uses selective labeling of methyl groups in leucine and isoleucine side chains to directly probe methyl−π contacts, one of the most prominent forms of interaction between proteins and small molecules. Using simple NMR chemical shift perturbation experiments with selected BRD4-BD1 binders, we find good agreement with a commonly used model of the ring-current effect as well as the overall interaction geometries extracted from the Protein Data Bank. By combining both interaction geometries and chemical shift calculations as fit quality criteria, we can position dummy aromatic rings into an AlphaFold model of the protein of interest. The proposed method can therefore provide medicinal chemists with important information about binding geometries of small molecules in fast and iterative matter, even in the absence of high-resolution experimental structures.

## Introduction

Modern drug discovery campaigns often start with the screening of small molecule libraries in order to identify an initial starting point for the subsequent optimization of binding affinities to a target of interest. Fragment-based drug discovery (FBDD), in which simple molecules are used in the screening process, has become especially popular as they offer significant advantages.^1^ For one, fragments cover the chemical space more efficiently than larger compounds, especially when care is taken to ensure chemical diversity of the used library.^2^ While this also means that fragments generally show fewer interactions and lower affinities, these interactions are often fundamental for target recognition and could otherwise be masked by unfavorable contacts in larger lead compounds.^3^ While interactions of polar groups like the formation of strong hydrogen bonds and electrostatic interactions dominate energetically favorable binding events for fragments, the majority of small molecule ligands also show other directional interactions like arene contacts or hydrogen bonds involving CH-groups, and their influence tends to increase with the size of the ligand.^4^ The most prominent of these is the formation of weak hydrogen bonds between aromatic π-systems, commonly of a small molecule, as the acceptor and aromatic or aliphatic CH-groups as the donor.^5,6^

Nuclear magnetic resonance (NMR) has been an integral method for FBDD from the very start.^7^ Simple protein detected 2D NMR experiments can identify low-affinity binders and readily distinguish them from false positive hits.^8^ NMR is especially well suited to detecting the CH−π interactions mentioned above, as the additional local magnetic field induced by aromatic ring currents leads to a large anisotropic chemical shift change in surrounding nuclei that can be readily measured by standard NMR experiments. This serves not only as a reporter of CH−π interactions, but as we showed in a previous work, the magnitude of the measured shift change also correlates well with the binding energy of the respective interaction.^9^ By this, NMR provides indirect information about the binding contribution of individual interactions as well as direct structural information at atomic resolution that aids medicinal chemists in their efforts to refine initial fragment hits into high-affinity binders. The potential impact is further emphasized by the relative frequency of aliphatic and aromatic amino acids in binding pockets as well as the abundance of aromatic ring systems in small molecule ligands.^6,10^ NMR does not come without its own set of limitations, namely spectral overlap and limited experimental sensitivity.^10,11^ To overcome these challenges, selective labeling strategies for specific residues and side chain atoms have been established which reduce spectral crowding and allow the use of optimized measurement schemes to utilize the favorable relaxation characteristics of certain chemical groups.^12,13^ In combination, this has opened up NMR-based FBDD approaches to high molecular weight proteins and complexes.^14^

In this work, we aim to extend the work we have previously conducted on tryptophane CH-groups toward the more accessible methyl groups of aliphatic amino acids, here namely, leucine and isoleucine. These are highly abundant in the hydrophobic cores of proteins as well as in the binding pockets for small molecules which are commonly found in less exposed sites of the protein which makes them valuable reporters of ligand binding events.^4^ The ease of interpretation of NMR experiments using selectively labeled side chains and the exquisite sensitivity of the chemical shift for changes in methyl−π interaction geometry allows for direct probing of these interactions.

To demonstrate the method, we use bromodomain 1 of bromodomain-containing protein 4 (BRD4-BD1) as our protein target. BRD4-BD1 acts as a chromatin reading protein by binding to acetylated histones and is therefore crucially involved in the regulation of protein expression.^15^ The highly conserved substrate binding site of BRD4-BD1 selectively recognizes acetylated Lysines of H4 histones and multiple potent inhibitors of this interaction have been developed in the past.^16,17^ Additionally, the binding pocket contains multiple leucine and isoleucine residues. Therefore, it acts as an ideal model system for our approach.

## Results and Discussion

### Selective Labeling

**Figure 1:**
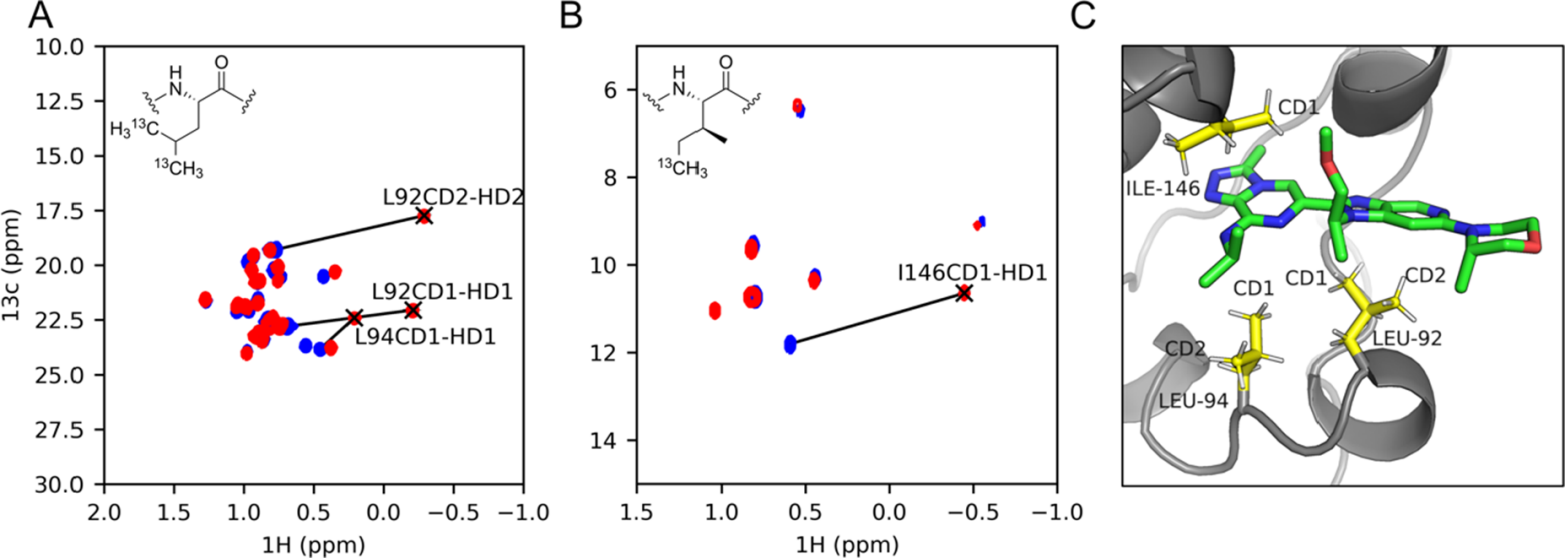
Overlay of ^1^H–^13^C HSQC spectra of selectively leucine (A) and isoleucine (B) labeled apo BRD4-BD1 (blue) and in complex with ligand 1 (red). (C) Position of ligand 1 and surrounding leucine and isoleucine residues (PDB: 6XUZ).

Selective labeling of the δ methyl groups of leucine and isoleucine residues was achieved by the addition of selectively labeled alpha-keto-acids as metabolic precursors to the *Escherichia coli* expression medium. In detail, 2-ketoisocaproate and 2-ketobutyrate were used to label leucine and isoleucine residues, respectively.^18,19^ In both cases, the δ position methyl carbons were selectively ^13^C–^1^H labeled and all other nonexchangeable protons in the side chain were deuterated to eliminate through bond coupling effects. In addition to reducing spectral crowding, the use of these precursors also improves signal-to-noise ratio by preventing intraresidue dipole–dipole-induced spin relaxation. We investigated a total of 16 ligands, 10 of which have already been described in our previous work on CH−π interactions^9^ (SI, Table 1).^9^ In total, BRD4-BD1 contains 13 leucine and 7 isoleucine residues. Of these, 2 leucines at positions 92 and 94 as well as one isoleucine at position 146 are present in the binding pocket and consequently experience large shift changes upon binding (Figure 1).

### Geometric Model of Methyl−π Interactions

**Figure 2:**
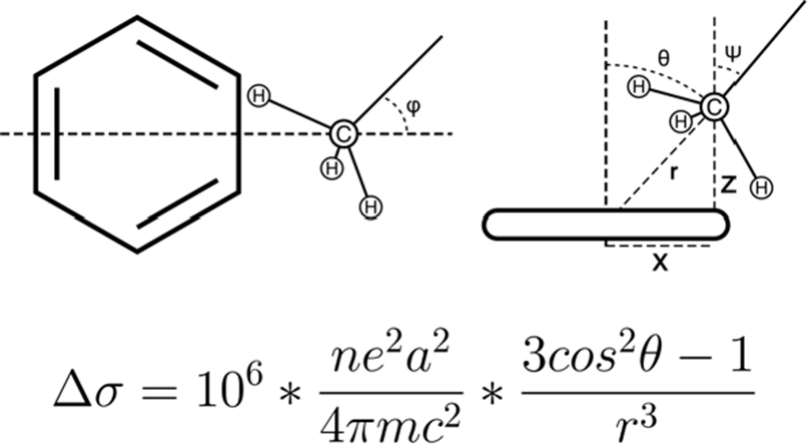
Top: Geometric model that unambiguously describes the position and orientation of a methyl vector relative to an aromatic ring system. Bottom: Pople point-dipole model was used to calculate the chemical shift change induced by a benzene ring.

To compare the measured chemical shift changes upon ligand binding to the ones expected, as calculated from available X-ray structures of the complexes, we apply the commonly used dipole model for chemical shifts induced by aromatic systems first introduced by Pople (Figure 2).^20^

Δσ refers to the change in the isotropic nuclear shielding constant in parts per million (ppm). *n* is the number of circulating electrons and is fixed at *n* = 6 in our case. *e* is the elementary charge in Franklin, *m* is the mass of an electron in gram, and *a* is the diameter of the aromatic ring in cm. The diameter was chosen based on the presence of a 5- or 6-ring and multiring systems were separated into their set of smallest rings (SI, Table 2).

*r* is the distance between the nucleus in question and the aromatic ring center in cm. θ is the angle between the vector connecting the nucleus under investigation with the ring center and the ring normal pointing in the same general direction. Both parameters were extracted from X-ray cocrystal structures. While this is straightforward for methyl carbons, methyl hydrogens are generally not present in X-ray structures. In addition, due to the fast rotation of the hydrogens around a common C–C symmetry axis, they are chemically equivalent, and their signals collapse to a single average over the three atoms. To address this, a more sophisticated geometric model must be used (Figures 2 and S1). In this model, we treat the methyl carbon not as a point in space but as the origin of a unit vector along the C–C symmetry axis of the methyl hydrogens (methyl vector). We get two such vectors for leucine (Cδ_1_/Cδ_2_ to Cγ) and two in isoleucine (Cδ_1_ to Cγ_1_ and Cγ_2_ to Cβ). This leads to a new set of four parameters *r*, θ, φ, and ψ that uniquely describe the vector’s position and orientation relative to the center and normal of an aromatic ring plane.

**Figure 3:**
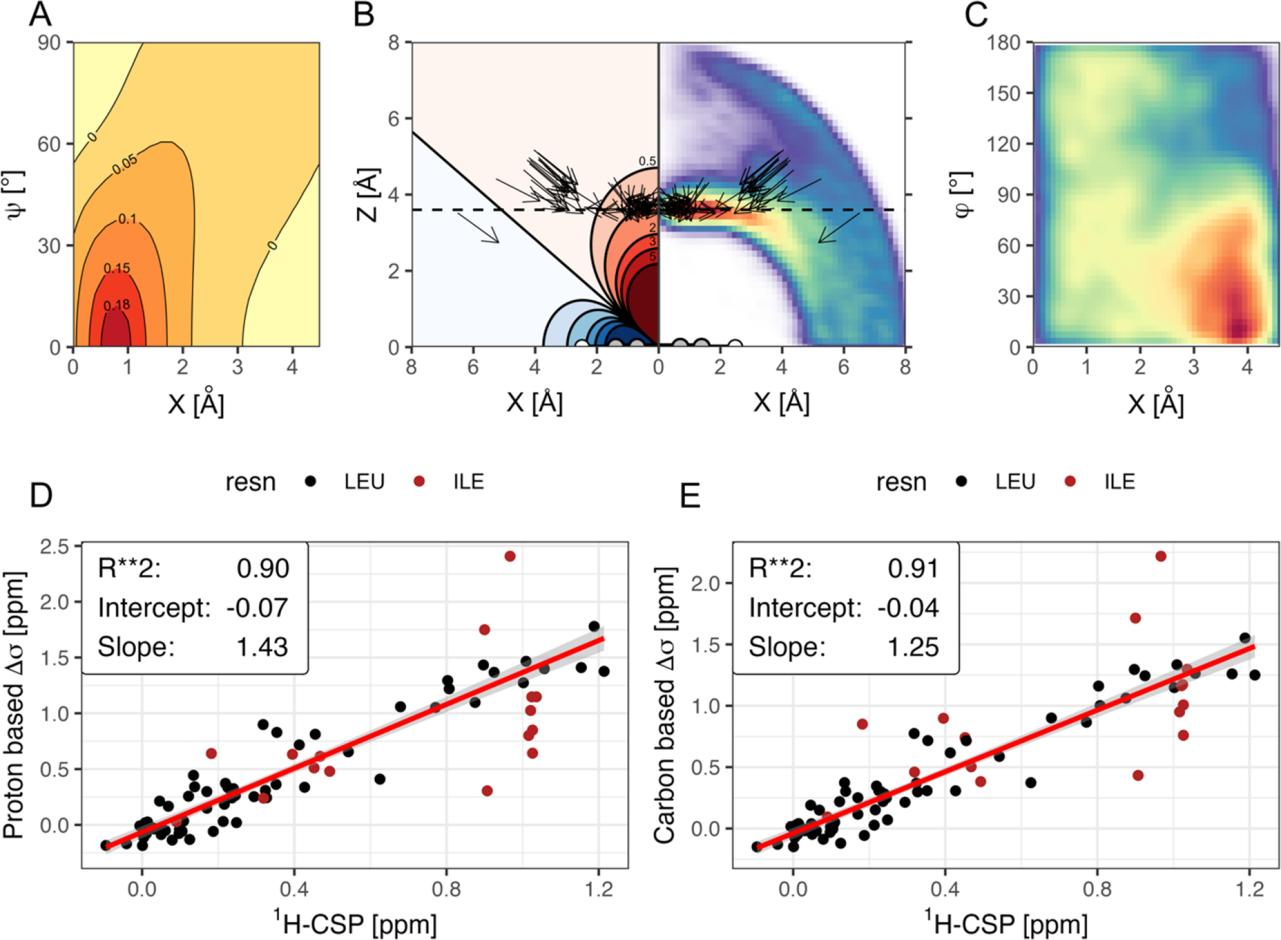
(A) Difference between the proton- and carbon-based models dependent on ψ angle and X-displacement along the ring plane at a constant normal distance (*Z*) for the methyl carbon of 3.8 Å and averaged over all φ angles. (B) Projection of the extracted methyl vectors (C–C symmetry axes) for all ligands under investigation above the aromatic ring plane. The colored areas indicate the expected Pople values (left) and the relative frequency of Leu methyl carbons in the PDB at the same position (right). (C) Frequency of φ values dependent on X-displacement along the ring plane. (D/E) Correlation between measured and calculated chemical shift changes for the leucine (black) and isoleucine (red) methyl groups. At this stage, the linear fit is based only on leucine shifts. Shifts are calculated either based on an average over ideal positions for the methyl protons (D) or the proxy position of the methyl carbons (E).

The parameters *r* and θ keep their previous meaning. In addition, we extracted parameters φ and ψ. In this case, φ is the angle formed between the respective projections of the methyl vector and the vector connecting the methyl carbon with the ring center onto the aromatic plane, and ψ is the angle between the methyl vector and the ring normal. By treating the aromatic ring as a simple plane, it holds no directional information, except for the ring normal. Therefore, the domains of both φ and ψ are limited to [0, π]. To reconstruct the expected position of the methyl hydrogens from this set of parameters, one can simply scale the methyl vector to the length of a CH–sp^3^ bond (1.087 Å) and rotate it around the vector resulting from the cross product of the methyl vector and the ring normal, centered at the methyl-carbon position, by 109.5° toward the aromatic ring plane. This vector can then be rotated in discrete steps around the original methyl vector. For each step, we extract new *r* and θ for that respective hydrogen position. By averaging the chemical shifts calculated via the Pople model for each step, we get the approximate chemical shift expected for the methyl hydrogens when rotated around their symmetry axis (Figure S6). When comparing the calculated proton chemical shift changes based on this proton model with the values we get when simply using the carbon position as a proxy (carbon-based model), the biggest differences arise close to the ring center at low ψ angles (Figure 3A). While methyl-carbon positions seem to be a good proxy for the averaged methyl proton center, carbon shifts themselves are not discussed in this paper as they are heavily influenced by additional factors like side chain rotamers among others that dominate over the ring-current effect.^21^

The geometric parameters extracted from the protein–ligand X-ray structures are visualized in Figure 3B. The black arrows represent the position of the methyl vectors above the ligand rings, with the arrow tips centered on the methyl-carbon position. In this projection of the 3-dimensional geometry, the angle ψ is indicated by the pitch of the vector. Increasing yaw angles φ in turn are represented by shortened arrows as the methyl vector is rotated out of the projection plane. The methyl carbons are predominantly positioned above the ring plane at a normal distance of 3.6 Å (Figure 3B, dotted line) and a slight displacement from the ring center.^22^ This is also the area in which one can expect a chemical shift change of around 1 ppm for the carbon atoms while the chemical shift change for the methyl protons will be averaged by their rapid rotation around the methyl axis, and plotting their expected shift becomes dependent on both φ and ψ (Figure 3B left). The same preferred position for methyl-carbon atoms above aromatic rings can also be extracted from the Protein Data Bank^23^ (PDB) as indicated by the heatmap in Figure 3B (right). It is apparent that while the pitch angle ψ takes up values around 60–70°, the yaw angle φ is either unconstrained above the center of the ring or very low beyond the ring diameter in order to point the methyl toward the ring center (Figures 3C and S4).

### Measured vs Calculated CSPs

We measure the experimental chemical shift change for BRD4-BD1 induced by the addition of a ligand from a set of 16 known binders (Table S1) and compare them to the expected shift change calculated from protein–ligand cocrystal structures. Upon ligand binding, we can detect large shifts for the δ-methyl resonances of BRD4-BD1 Leu 92, 94, and Ile 146 (Figure 1). The correlation between measured chemical shift changes induced by ligand binding versus the expected values calculated using the Pople model is shown in Figure 3D,E. For Leu-δ methyl groups, both the carbon proxy model (Figure 3E) as well as the average over all possible proton rotamers (Figure 3D) show similar *R*^2^-values of 0.90 and 0.91, respectively. The main difference between the two models is the deviation from the expected slope of 1, which is more pronounced for the proton model. The reason for this deviation is the lack of proper parametrization of the Pople model depending on the nature of the aromatic ring.^24,25^ As the Pople model is originally parametrized for a benzene molecule, a ring-current intensity factor is commonly fitted as a scaling factor that relates the expected intensity in benzene to the one in the ring under investigation.^24^ The carbon proxy model seems to be a good approximation for small changes in binding geometries that are averaged in solution which are not reflected in a static X-ray structure, compared to the proton model, as it is less sensitive to small changes in φ and ψ angles (Figure 3A) that are sampled in solution.

### Outlier Correction

**Figure 4:**
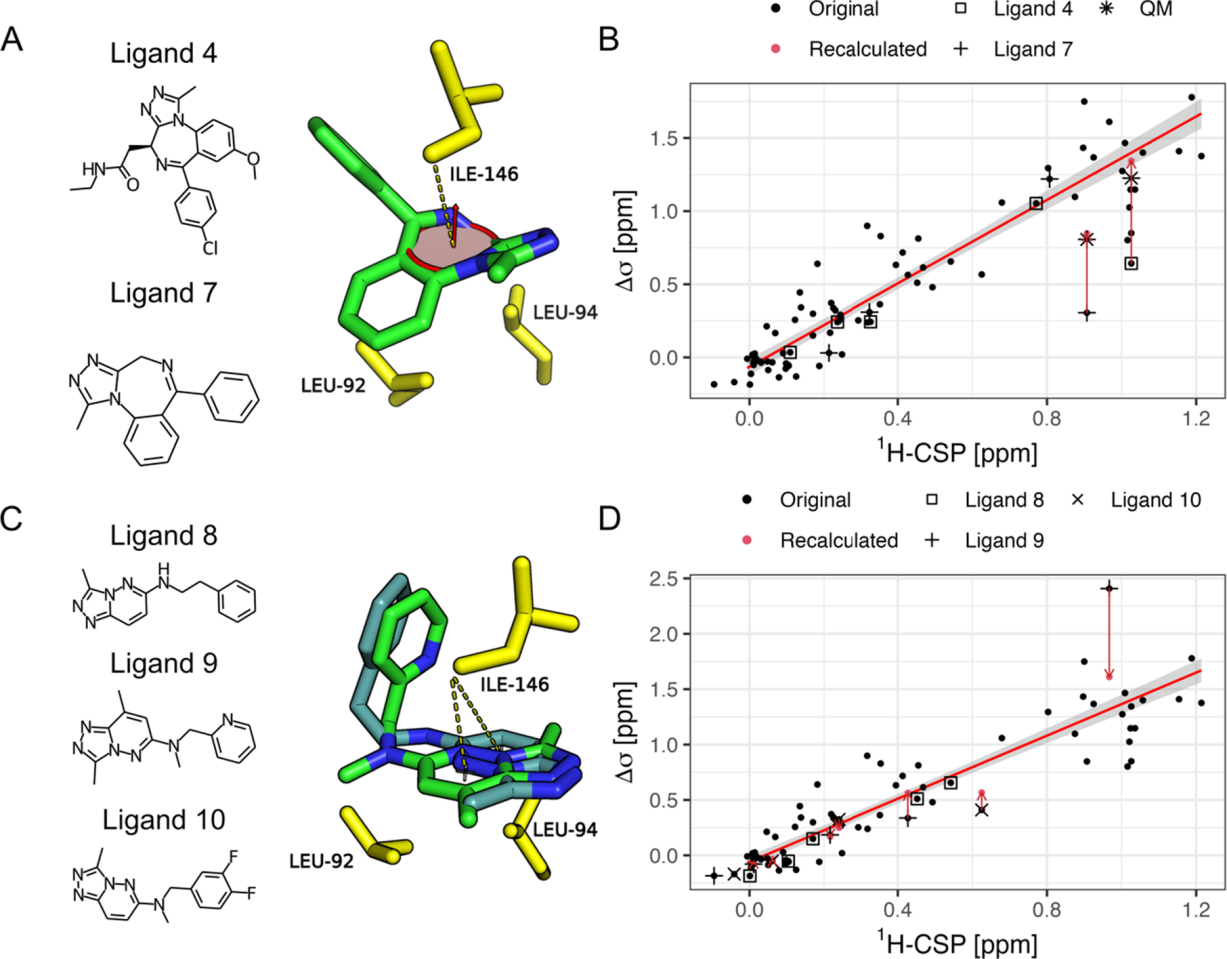
(A) X-ray structure of ligand 7. The central nonplanar diazepine ring found in ligands 4 and 7 can be converted to a consensus plane over all ring atoms in order extract the relevant parameters for the Pople model. 2D structures of ligands 4 and 7 are depicted in ascending order. (B) Correlation of calculated isoleucine shifts for ligands 4 and 7 is improved by the homoaromatic ring approximation (red arrows). QM-based shift calculations predict similar values after being scaled by the slope of the linear fit over all of the leucines. (C) Overlay of ligands 8 and 9. The aromatic polycycle shared among ligands 8 and 9 is rotated in ligand 8 around one of the ring centers which places the Ile-Cδ_1_ directly above the other ring center. 2D structures of ligands 8, 9, and 10 are depicted in ascending order. (D) Calculated shifts for ligand 9 reflect measured data (red arrows) when they are calculated from an average over the ring position for ligands 8 and 9.

The biggest discrepancy between measured and calculated values is present for the signals measured for Ile146 in BRD4-BD1. For ligands 4 and 7, the Ile-δ_1_ methyl group under investigation is positioned in the vicinity of a nonaromatic diazepine ring. In contrast, the leucine methyl groups are positioned above aromatic 5- and 6-rings and consequently fit the data. The 7-membered diazepine ring may experience through space conjugation of its p-orbitals leading to a weak homoaromatic ring-current effect, that in turn affects the methyl proton shifts of Ile146 upon ligand binding.^25,26^ The Pople model is not able to deal with systems like this and, therefore, underestimates the chemical shift change. As an approximation of the homoaromatic effect, a consensus plane was created based on the diazepine-ring atoms (Figure 4A). When the geometric parameters for the Pople model are extracted based on this plane, the agreement between experimental and calculated shifts for Ile146-δ1 improves drastically (Figure 4B). To test the validity of this approximation, we performed quantum mechanics (QM) based calculations of the bound and unbound shifts for ligands 4 and 7. The resulting theoretical CSPs can reproduce the experimental values and, after scaling by the slope of the linear fit, align well with our diazepine-ring pople approximation. (Figure 4B).

**Figure 5:**
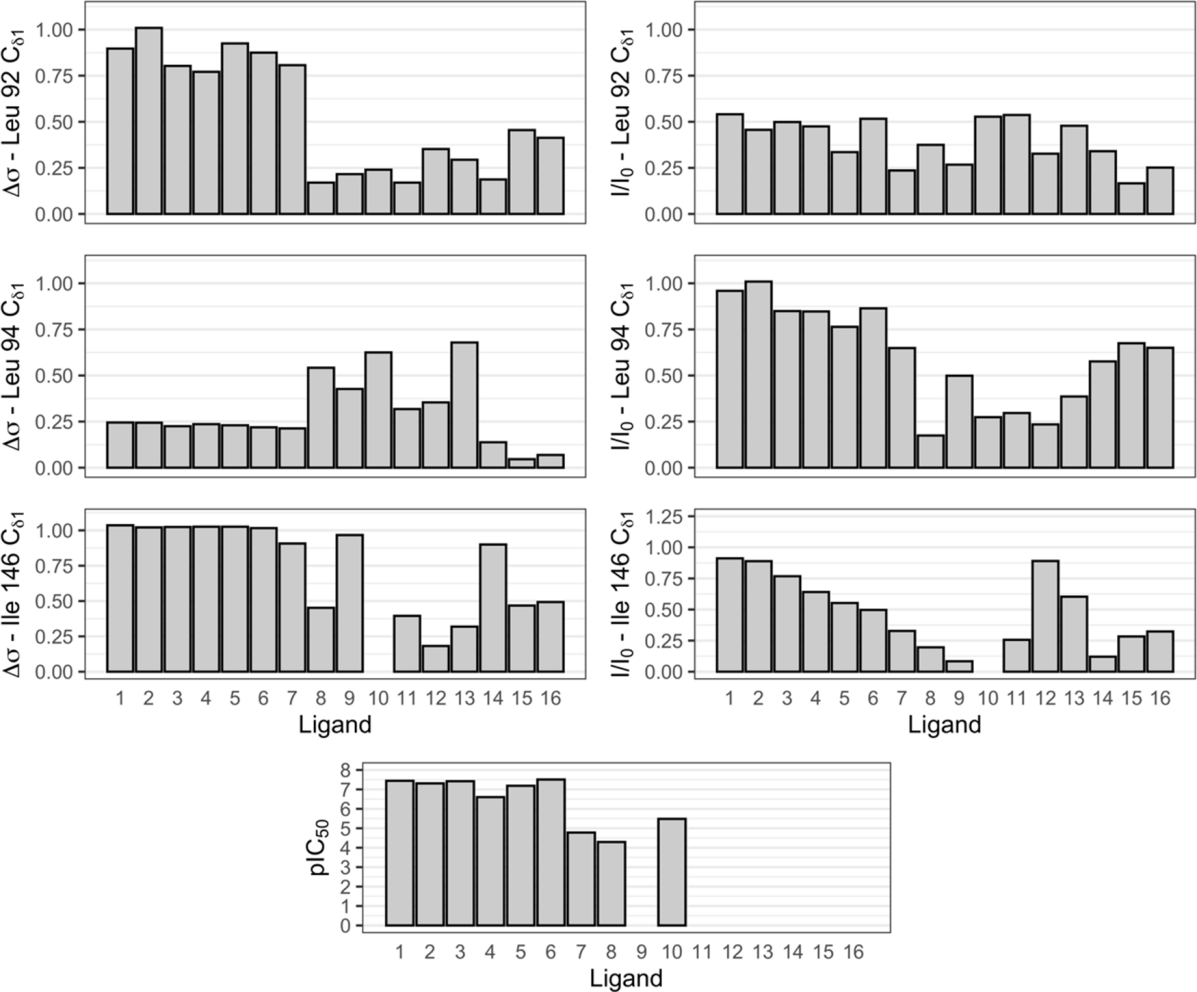
Chemical shift changes and intensity ratios between free and bound state for Cδ1 methyl groups as well as AlphaScreen^27^ pIC50 values. For ligands 9 as well as 11–16, no affinity information is available as they were fragment hits that yielded X-ray co-structures but for which no *K*_D_ values were determined. Intensity ratios for leucine 92 Cδ1 are reduced due to partial overlap of the apo state (Figure S3).

For ligand 9 in turn, the calculated shift for the Ile-δ_1_ methyl group is much larger than the experimental value. Upon closer inspection, ligand 9 shares a central substructure with ligands 8 and 10. In the corresponding X-ray structures, it is apparent that the 5,6-ring substructure for ligand 8 is rotated around the center of the 5-ring compared to ligands 9 and 10. This leads to a major change of the methyl−π geometry between the Ile-δ_1_ and the 6-ring center (Figure 4C). A plausible explanation for the overestimation of the calculated shift in this case is that the rings of ligands 9 and 10 also sample the corresponding positions occupied by ligand 8 and exchange between both states on a medium to fast time scale. Further support comes from the fact that these 3 ligands are also the ones showing the largest signal attenuation for the Ile-δ_1_ signal upon ligand binding, in the case of ligand 10 even beyond detection (Figure 5). For all 3 of them, crystallographic B-factors for the Cδ_1_ positions as well as the average over the respective ring atoms are increased compared to, e.g., ligand 1 (Table S2). This further suggests conformational exchange between both states. If the shift changes for ligand 9 are recalculated from an average of the ring position for ligands 8 and 9, then both the isoleucine and leucine shifts improve significantly (Figure 4D).

### Shift Patterns and Relative Intensity

The intensity ratio of the respective side chains indicates additional flexibility of the methyl groups, alternative binding modes of the ligand, or exchange between free and bound states, all of which indicate an opportunity for Medicinal Chemists to increase binding affinity further, by pointing to the individual molecular interaction that is not optimal yet. Here, especially Ile146 is of interest as we observe a wide range of intensities from completely exchange broadened to no decrease compared with the apoprotein. Inspection of the intensity ratios in our set indicates that a stable binding mode was achieved over the course of compound optimization for high-affinity compounds.

In contrast to Ile146, for Leu94 the change in signal intensity in most cases seems to be dependent on the presence of an additional fused ring system in the ligand, which leads to less exchange broadening of Leu94. This suggests that the broadening experienced by Ile146 and Leu94 is not due to the global dynamics of the protein but rather the residual mobility of the ligands in the binding site. While the relative signal for Leu92 seems to be reduced for all ligands, the true effect is hard to gauge as there is peak overlap for the apo signal (Figure S3). While the magnitude of the chemical shift changes upon binding does not directly report on the affinity of the overall interaction, it does indicate which methyl groups are addressed by the aromatic ring systems. This is especially clear when comparing the ligands with either 1 (8–16) or 2 (1, 2, 3, 5, 6) fused ring systems in the set. In the former case, mostly Leu94 and Ile146 are addressed, while the addition of an additional ring system suggests a reduced interaction with Leu94 in favor of both Leu92 and Ile146.

### Ring Fit Based on Shift and the PDB

**Figure 6:**
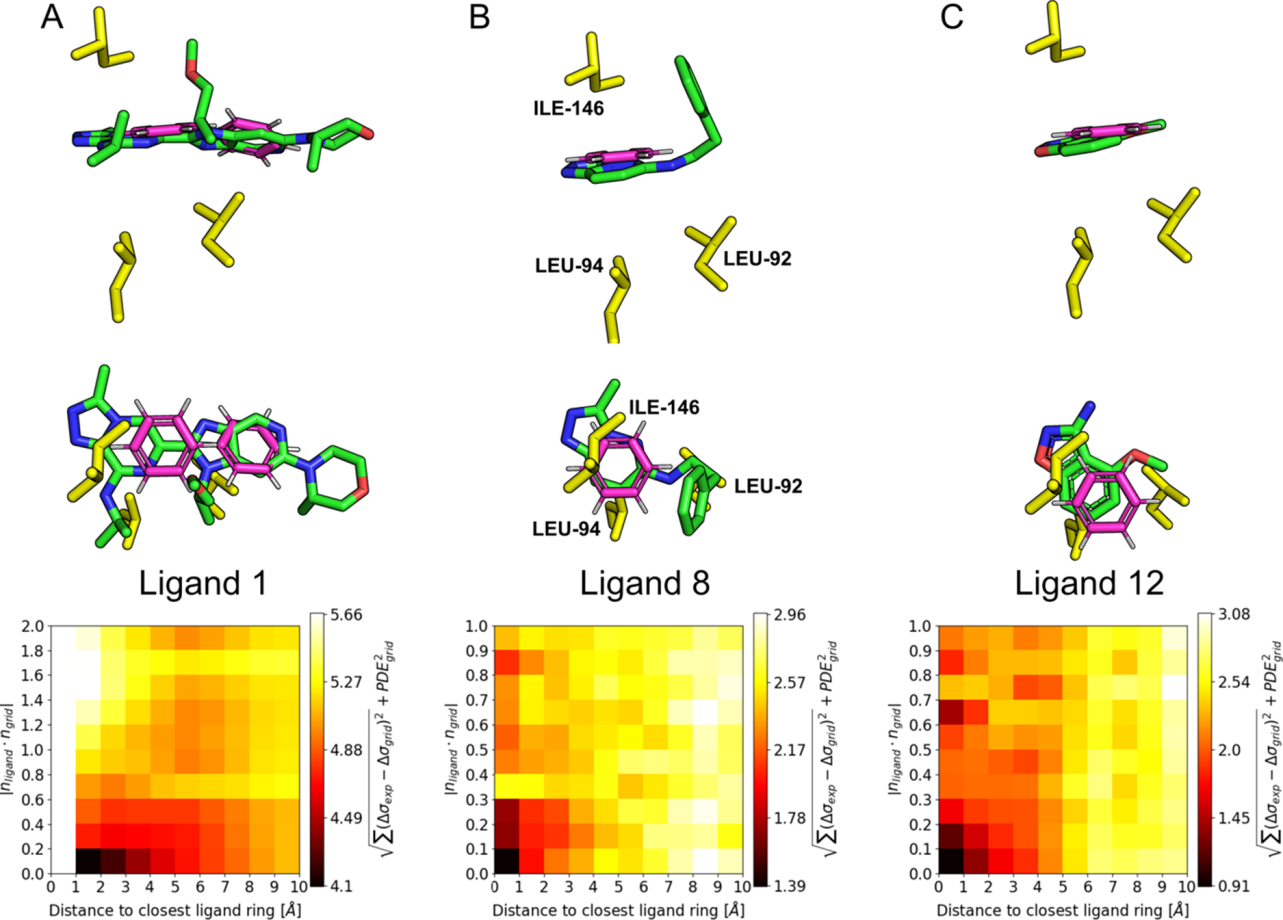
Examples for ring location and orientation fit based on methyl shifts and interaction geometry probabilities. Side and top views of the bound ligand structures overlaid with the best ring fits in magenta (top) and average fit value dependent on closest ligand ring position and orientation (bottom). Each cell of the heatmap represents the average fit value at the respective difference in position and orientation (absolute cosine value of the angle) of the closest aromatic ring of the ligand crystal structure. For all examples, we find the lowest fit values close to those of the true conformation of the aromatic rings. (A) Two aromatic rings must be fit simultaneously in order to cover both fused ring systems of ligand 1 (PDB 6XUZ). Due to the grid spacing, the combined distance to the closest ring is larger than 1 and the heatmap cells below 1 Å are therefore empty. (B, C) For ligands 8 (PDB entry 9FWX) and 12 (PDB entry 9FXP), a single aromatic ring is sufficient to find the correct position and orientation.

In the early 2000s, McCoy et al. introduced the concept of *j*-surfaces in which they used backbone NH shifts induced by aromatic rings to locate the origin of the perturbation and therefore the most probable position of aromatic ligand rings.^28^ As the methyl groups in our examples are directly interacting with the aromatic rings compared to the mostly indirect effects experienced by backbone NH atoms, as is apparent from the large magnitude of the induced shift, it should also be possible to fit the position and orientation of aromatic rings in our case. For this purpose, we examined the agreement between experimental and calculated shift perturbations induced by a single benzene ring within a grid of ring origin locations and ring normal orientations (Figure 6). In addition, to compensate for the rotational degeneracy of the simple Pople dipole model, we also incorporate the probability for the interaction geometry parameters (*r*, θ) as extracted from the PDB (Figures 3B and S7–S10).

For a single or fused aromatic ring system in the ligand, one benzene ring is sufficient to find the correct position and orientation of the ring responsible for the largest effect (Figure 6B,C). When a second fused ring system is present, a second benzene ring is necessary to find a good agreement between experimental and calculated values (Figure 6A). In practice, the necessity of a second ring can be inferred from both the structure of the ligand as well as the chemical shift perturbation pattern induced by ligand binding. In all examples, the smallest fit values are found for grid positions resembling the experimental structures. If the correct assumptions are made, we find ring conformations close to the experimental ones for our full set of ligands (Figure S11) As we have used a structure provided by AlphaFold^29,30^ in all the fitting examples, this approach is available even in the absence of an experimentally determined apo-structure and is robust for minor rearrangements of side-chain conformations upon binding. Apart from finding the most probable origin of the induced chemical shift as described above, one straightforward application is to evaluate and rank ligand docking poses based on the measured chemical shifts as well as the methyl-ring geometries.

## Conclusions

FBDD relies on the initial discovery of weak binding ligands and subsequent improvement of noncovalent interactions between said ligands and the protein target. At both stages, NMR can serve as a valuable tool due to the sensitivity of the chemical shift, even to small variations of the chemical environment on an atomic level. One especially prominent form of interaction between proteins and their small molecule ligands are CH–hydrogen bonding interactions, involving aromatic ring systems and both sp^2^- and sp^3^-hybridized carbons, commonly termed CH−π interactions. Though they are weak individually, they serve to fine-tune the overall intermolecular interaction and consequently have a big impact on both affinity and selectivity. In this work, we show that one can directly detect and quantify methyl−π interactions even in the absence of a protein–ligand cocrystal structure. The binding site can be derived on a sequence level from the residue-specific experimental chemical shifts, while the contribution of methyl−π interactions can be gauged from the magnitude of the chemical shift change. The use of selectively labeled amino acid precursors leads to increased signal-to-noise levels and, if needed, a residue-specific simplification of the resulting methyl spectra.

While there are some discrepancies between the measured and expected chemical shift values, these can easily be attributed to increased flexibility of side chains and ligands as well as to the presence of homoaromatic groups that, while not being explicitly covered by the Pople model, can be approximated to a satisfactory degree as we demonstrated. The observed differences that can be attributed to the lack of ring parametrization follow a linear relationship and can therefore be estimated as a simple scaling factor from measuring multiple methyl positions and, if necessary, different but structurally related ligands.^31^

We further showed that there is a specific interaction geometry for methyl−π interactions both in our ligand set as well as in the PDB. The distinct orientational propensity of methyl groups relative to aromatic rings, in combination with the exquisite dependence of the proton chemical shift on these orientations, allows one to reliably determine or extract protein–ligand geometries.

The combination of the binding geometry probabilities extracted from the PDB and the simple chemical shift model to fit the position and orientation of aromatic rings from a small set of methyl shifts dramatically improves the fit. Positioning of the dummy aromatic centers should in many cases resolve the binding site and conformation of the ligand aromatic groups if they interact directly with a methyl group of the protein.

As the computation time to calculate the chemical shift is negligible compared to more sophisticated QM and density functional theory methods, this approach provides fast iterative feedback for medicinal chemists in the process of fragment refinement on the interaction geometry of crucial methyl−π interactions and allows for a more informed decision-making approach, especially as the relative intensity of the measured signal provides insights into residual mobility of the ligand in the binding pocket and therefore indirectly reports on binding energies as demonstrated within our set of BRD4-BD1 ligands.

Additional labeling strategies already presented or in development make additional aliphatic groups available for use with this approach.^32,33^ In the future, we aim to be able to compile these into a novel and comprehensive refinement approach that can unanimously probe the geometries of CH−π interactions in protein–ligand binding sites and subsequently determine the complex structures at unprecedented speed. Due to the prevalence of aromatic centers in both small fragments and larger, highly optimized ligands, this will allow identification and evaluation of docked conformation at all stages of development.

## Experimental Section

### Ligand Synthesis

Ligands 1–10 were synthesized according to Platzer et al.^9^ and are >95% pure by high-performance liquid chromatography (HPLC) analysis (Figures S12–S21). Compounds 11–16 were purchased from commercial vendors and tested without additional HPLC control.

### Protein Expression and Purification

Human BRD4-BD1 (44–168) containing a TEV-cleavable His6-tag was overexpressed in *E*. *coli* BL21(DE3). 4L of bacteria were grown to an optical density of 0.6. After pelleting (20 °C) and resuspension in 1L of M9 minimal medium, expression was induced with a final IPTG concentration of 0.4 mM and continued overnight. After harvesting, the cells were resuspended in 30 mL of phosphate buffer (20 mM sodium phosphate, 500 mM NaCl, 20 mM Imidazole, pH 7.5), sonicated, and subsequently loaded onto a Ni^2+^ affinity column. After step gradient elution and buffer exchange of the protein-containing fractions under low imidazole conditions, the protein was subjected to TEV-cleavage overnight (4 °C). His6-TEV protease and cleaved his6-tag was removed by a second pass over a Ni^2+^ affinity columns. After subsequent buffer exchange (10 mM sodium phosphate, 100 mM NaCl, pH7.5) and concentration to 0.3 mM using a 3kD cutoff centrifugal filter device, the protein was stored at −20 °C.

All samples were uniformly ^15^N-labeled. To achieve selective labeling of leucine and isoleucine δ positions, either 2-ketoisocaproate or 2-ketobutyrate was added at a final concentration of 100 mg/L to the minimal media prior to induction by IPTG. All precursors used in this work were provided by MAG-LAB.

### NMR Measurements and Analysis

All protein NMR measurements were carried out at 298 K on a Bruker Avance HD3+ 600 MHz spectrometer. 2D ^1^H–^13^C HSQC spectra were acquired using the pulse sequence “hsqcetgpsi” of the Bruker library^34−36^ using 64 (*t*_1_) × 512 (*t*_2_) complex points with acquisition times of 16.9 ms (*t*_1_) and 53.2 ms (*t*_2_). Assignment of Leu-δ methyl groups was achieved by mutational analysis. Four BRD4 mutants were produced selectively labeled (V87A, V90A, L92A, L94A) to assign resonances of those key residues in the binding site (Figure S2). Worth noticing here is that the mutation to an alanine leads to subtle rearrangements in the conformation of different neighboring residues, which in turn leads to chemical shift perturbations of other methyl resonances. Thus, in our case, unambiguous assignment of the four residues of interest (V87, V90, L92, L94) was only possible after the acquisition of spectra of all four mutants.

Stereospecific assignment of the two diastereotopic methyl groups of leucine and valine residues was achieved by fractional ^13^C-labeling described previously.^37^

All samples contained a final protein concentration of 0.2 mM, and a final ligand concentration of 1 mM.

### PDB Statistics

To generate statistics about distance and angle distributions between methyl groups of leucine residues and aromatic rings of ligand molecules, all X-ray structures in the PDB with a resolution smaller than 2.5 Å and a r-free smaller than 0.24 were used. All nonpolymer residues were treated as ligands and the parameters for all aromatic rings of this set were extracted as well as the parameters for all Leucines found in polymeric sequences. To reduce the redundancy of the set, the protein sequences were clustered into groups with a sequence similarity ≥95%. Consequently, for each leucine, a new sequence number was assigned representing its position in a multiple sequence alignment of all cluster members. The set was then further filtered to contain only the one leucine with the smallest distance between methyl carbon and ring center for each combination of cluster ID, cluster sequence number, and ligand name.

The kernel density estimation (kde) visualizations in Figure 3A–C were produced in R using the “kde” package with a grid size of 50 points in both dimensions.

### QM Chemical Shift Calculations

Calculations were performed for the structures of ligands 4 and 7 as well as the APO form. The systems consisted of Leu92, Leu94, and Ile146, along with the side chains of residues located within a 4 Å distance from the methyl groups of interest, namely Pro82, Val87, Cys136, Tyr139, and Asn140.^38^ In the case of HOLO forms, the ligands were also included. For leucine and isoleucine residues, the N- and C-termini were capped as amides with the preceding carbonyl carbon and with the following amino group, respectively. The remaining residues were truncated at the Cβ atoms. The systems were completed with dummy hydrogen atoms.

Chemical shieldings were calculated using the gauge independent atomic orbital (GIAO) approach, at the wB97XD/def2-TZVP level of theory, as implemented in the Gaussian16 package.^39−41^ To determine the chemical shift perturbations (CSPs), we subtracted the chemical shielding values of the APO form from those of the HOLO form.

### Fit of Ring Positions

The structure of apo BRD4 was downloaded from AlphaFold^29,30^ (Uniprot: Q5BJ26, version: 2022–11–01) and shortened to include residues 42–168. A grid representing potential ring centers was constructed around the geometric center of Cδ_1/2_ atoms of leucine 92 and 94 as well as the Cδ_1_ atom of isoleucine 146 with uniform dimensions of 10 Å and a grid spacing of 1 Å. This grid is further simplified by removing positions that are within 3 Å of any non-hydrogen atom in the structure. To sample the potential orientations of the ring normal, the φ and ψ spherical coordinates of the vertex positions of a once subdivided icosahedron are extracted. As parallel normals are equivalent due to the squared cosine term of the Pople equation, φ and ψ ranges of [0, π] and [0, π] are sufficient to effectively sample the complete icosphere. This leads to a final grid with dimensions 11 × 11 × 11 Å and 22 orientations. For each grid point, the distance *r* and angle θ are calculated for each above-mentioned methyl-carbon position, and the root of the squared difference between the experimentally measured CSP and the Pople model is calculated. If two rings are to be sampled simultaneously, the combination of each grid position with all others is evaluated, and combinations that are within 3.5 Å of one another are excluded.

To incorporate the probability of different methyl−π geometries, a two-dimensional Gaussian kernel density estimation (kde) was constructed based on the distance and θ angle between methyl carbons and aromatic rings in the PDB. The kde was constructed separately for Cδ_1/2_ positions of leucine and Cδ_1_ positions of isoleucine and weighted by 1/4 × π × *r*^2^. The kde probabilities are subsequently rescaled by subtracting them from the maximum value. Just as before, for each grid position, the kde is evaluated for each methyl-carbon position and the probabilities are summed up.

The delta shift root of squared differences and the squared kde probabilities are summed up to a single final fit value, and the grid position of the minimal value is reported as the expected ring position and orientation. Even small deviations from the true position and orientation of aromatic rings in the crystal structure lead to a major increase in the fit value both for the summed value and for its single components (Figures S7–S9B–D).

## Supporting Information Available

Compound structures, molecular formula strings, measured and calculated chemical shift values, B-factor comparison, Pople model coefficients, interaction geometry model, protein methyl assignment information, partial peak overlap, relative methyl ψ-X frequency in the PDB, top-down methyl vector projection, stepwise proton based chemical shift calculations, ring fit statistics, HPLC traces (PDF)
Molecular_formula_strings (CSV)
New PDB IDs: 9FWX and 9FXP. Authors will release the atomic coordinates upon article publication.

## Notes

The code used to extract interaction geometries from the PDB and fit the dummy ring positions is available upon reasonable request.
