# Supplementary material for: Probing Protein–Ligand Methyl−π Interaction Geometries through Chemical Shift Measurements of Selectively Labeled Methyl Groups
Source: J Med Chem. 2024 Jul 29;67(15):13187–96. doi: 10.1021/acs.jmedchem.4c01128 (PMC11320577; doi:10.1021/acs.jmedchem.4c01128)
Supplement: Supplementary file 1 — jm4c01128_si_001.pdf [file jm4c01128_si_001.pdf]

Supporting Information

Probing Protein-Ligand Methyl-π Interaction Geometries Through Chemical Shift Measurements of Selectively Labelled Methyl Groups

Andreas Beier[1,3,4*], Gerald Platzer[6], Theresa Höfurthner[1,3,4], Aleksandra L. Ptaszek[1,3], Roman J. Lichtenecker[2,6], Leonhard Geist[5], Julian E. Fuchs[5], Darryl B. McConnell[7] Moriz Mayer[5] and Robert Konrat[1,4]

[1] *Christian Doppler Laboratory for High-Content Structural Biology and Biotechnology, Department of Structural and Computational Biology, Max Perutz Laboratories, University of Vienna, Campus Vienna Biocenter 5, 1030 Vienna (Austria).*

[2] *Christian Doppler Laboratory for High-Content Structural Biology and Biotechnology, Institute of Organic Chemistry, University of Vienna, Währingerstraße 38, 1090 Vienna (Austria).*

[3] *Vienna Doctoral School of Chemistry, University of Vienna, Währingerstraße 38, 1090 Vienna (Austria)*

[4] *Max Perutz Laboratories, Department of Structural and Computational Biology, Campus Vienna Biocenter 5, 1030 Vienna.*

[5] *Boehringer Ingelheim RCV GmbH & Co. KG, Dr. Boehringergasse 5-11, 1121 Vienna.*

[6] *MAG-LAB, Karl-Farkas-Gasse 22, 1030 Vienna.*

[7] *Curie.Bio, 177 Huntington Ave Ste 1703, Boston, MA 02115-3153*

* *corresponding author Andreas Beier, andreas.beier@univie.ac.at*

Supporting Figures and Tables

Table S1 .... S2-5

Table S2 .... S6

Table S3 .... S6

Figure S1 .... S6

Figure S2 .... S7

Figure S3 .... S7

Figure S4 .... S8

Figure S5 .... S8

Figure S6 .... S9

Figure S7 .... S10

Figure S8 .... S11

Figure S9 .... S12

Figure S10 .... S13

Figure S11 .... S14

HPLC Traces S12-21 .... S15-20

Index	IC50 (uM)	Smiles	Structure	ILE-146-CD1			LEU-92-CD1			LEU-92-CD2			LEU-94-CD1			LEU-94-CD2		
				exp	calc	I/I0	exp	calc	I/I0	exp	calc	I/I0	exp	calc	I/I0	exp	calc	I/I0
**1**	0.036	COC[C@H](C)n1c(-c2cn3c(C)nnc3c(NC(C)C)n2)nc2cnc(N3CCOC[C@@H]3C)cc21	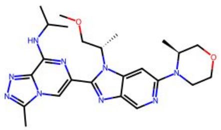	1.04	1.15	0.91	0.90	1.43	0.54	1.06	1.40	0.63	0.25	0.26	0.96	0.03	-0.04	1.01
**2**	0.049	COC[C@H](C)n1c(-c2cn3c(C)nnc3c(NC(C)C)n2)nc2ccc(C3CCOCC3)cc21	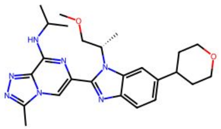	1.02	1.03	0.89	1.01	1.47	0.46	1.21	1.38	0.64	0.24	0.29	1.01	0.03	-0.03	0.97
**3**	0.038	Cc1nnc2c(NC(C)C)nc(-c3nc4cnc(N5CCOC[C@@H]5C)cc4n3[C@@H](C)c3ccccc3)cn12	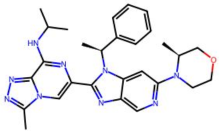	1.02	1.15	0.77	0.80	1.29	0.50	1.00	1.27	0.58	0.23	0.34	0.85	0.02	-0.01	0.93
**4**	0.249	CCNC(=O)C[C@@H]1N=C(c2ccc(Cl)cc2)c2cc(OC)ccc2-n2c(C)nnc21	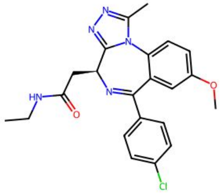	1.03	0.64	0.64	0.77	1.05	0.47	0.33	0.24	0.72	0.24	0.24	0.85	0.11	0.03	0.82

Index	IC50 (uM)	Smiles	Structure	ILE-146-CD1			LEU-92-CD1			LEU-92-CD2			LEU-94-CD1			LEU-94-CD2		
				exp	calc	I/I0	exp	calc	I/I0	exp	calc	I/I0	exp	calc	I/I0	exp	calc	I/I0
**5**	0.065	Cc1nnc2c(NC(C)C)nc(-c3nc4ccc(C5CCOCC5)cc4n3[C@@H](C)c3ccccn3)cn12	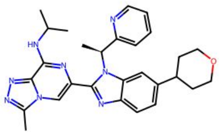	1.03	0.85	0.55	0.93	1.37	0.34	1.15	1.41	0.35	0.23	0.32	0.76	0.01	-0.01	0.51
**6**	0.031	Cc1nnc2c(NC(C)C)nc(-c3nc4ccc([C@H]5CN(C)CCO5)cc4n3[C@@H](C)c3ccccc3)cn12	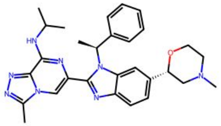	1.02	0.80	0.50	0.88	1.10	0.52	1.19	1.78	0.36	0.22	0.37	0.86	0.01	0.01	0.94
**7**	16.596	Cc1nnc2n1-c1ccccc1C(c1ccccc1)=NC2	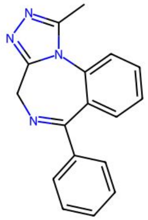	0.91	0.31	0.33	0.81	1.22	0.24	0.32	0.31	0.71	0.21	0.03	0.65	broadened		
**8**	50.926	Cc1nnc2ccc(NCCc3ccccc3)nn12	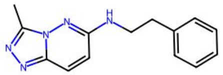	0.45	0.51	0.20	0.17	0.15	0.37	0.00	-0.19	0.59	0.54	0.66	0.17	0.10	-0.05	0.55

Index	IC50 (uM)	Smiles	Structure	ILE-146-CD1			LEU-92-CD1			LEU-92-CD2			LEU-94-CD1			LEU-94-CD2		
				exp	calc	I/I0	exp	calc	I/I0	exp	calc	I/I0	exp	calc	I/I0	exp	calc	I/I0
**9**		Cc1cc(N(C)Cc2ccccn2)nn2c(C)nnc12	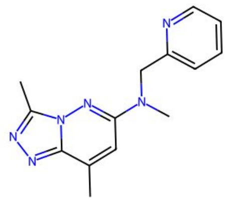	0.97	2.41	0.08	0.22	0.18	0.27	- 0.10	-0.18	0.83	0.43	0.34	0.50	0.01	-0.08	0.79
**10**	3.283	Cc1nnc2ccc(N(C)Cc3ccc(F)c(F)c3)nn12	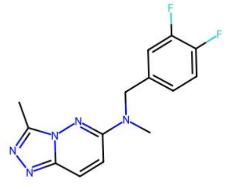	broadened			0.24	0.32	0.53	- 0.04	-0.17	1.49	0.63	0.41	0.27	0.06	-0.05	0.62
**11**		Cc1cc2c(N)cccc2nn1	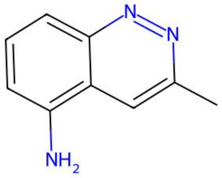	0.40	0.63	0.26	0.17	0.30	0.54	0.08	-0.14	0.88	0.32	0.90	0.30	0.05	-0.03	1.07
**12**		COc1cccc2onc(N)c12	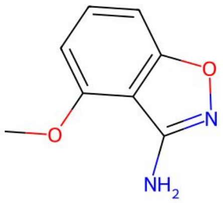	0.18	0.64	0.89	0.35	0.36	0.33	0.10	-0.08	0.79	0.35	0.83	0.23	0.01	-0.03	0.15

Index	IC50 (uM)	Smiles	Structure	ILE-146-CD1			LEU-92-CD1			LEU-92-CD2			LEU-94-CD1			LEU-94-CD2		
				exp	calc	I/I0	exp	calc	I/I0	exp	calc	I/I0	exp	calc	I/I0	exp	calc	I/I0
**13**		Cc1nnc2ccc(N3CCC(C)CC3)nn12	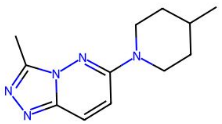	0.32	0.24	0.60	0.29	0.25	0.48	0.05	-0.09	0.75	0.68	1.06	0.39	0.11	-0.06	0.62
**14**		Cc1ccc(CNc2cc(C)nc3nncn23)cc1	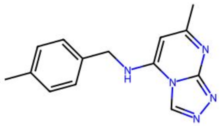	0.90	1.75	0.12	0.19	-0.06	0.34	0.13	-0.13	0.58	0.14	0.34	0.58	0.00	-0.11	0.15
**15**		CCN1C(=O)CCc2cc(NC(C)=O)ccc21	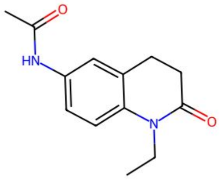	0.47	0.61	0.28	0.46	0.81	0.17	0.25	0.02	0.54	0.05	0.21	0.67	0.01	0.03	1.05
**16**		C[C@@H]1C(=O)N(C)c2cnc(N)nc2N1C1CCCC1	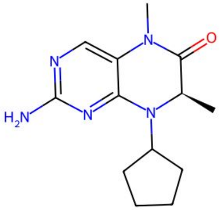	0.49	0.48	0.32	0.41	0.72	0.25	0.10	-0.04	0.84	0.07	0.17	0.65	0.01	0.02	0.93

Table S1: List of ligands with their AlphaScreen IC_{50} values, as well as the measured and calculated shifts based on the Pople model

	B factor			
Ligand	Leu 92 CD1	Leu 94 CD1	Ile 146 CD1	Ring average
Ligand 1	11.35	13.55	11.29	7.79
Ligand 8	14.22	16.08	11.42	11.81
Ligand 9	18.09	19.26	20.64	19.38
Ligand 10	13.58	11.99	20.24	15.11

Table S2: B-factors for selected atoms and structures. Ring average is the average over all ring atoms of the double ring directly beneath Ile-146-CD1

n	number of ring electrons	6
e	elemetary charge	4.8032×10^{-10} Fr
m	electron mass	9.1094×10^{-28} g
c	speed of light	2.998×10^{8} cm/s
a	ring diameter (5 ring)	1.18×10^{-8} cm
	ring diameter (6 ring)	1.39×10^{-8} cm

Table S3: Pople model parameters used in cgs units.

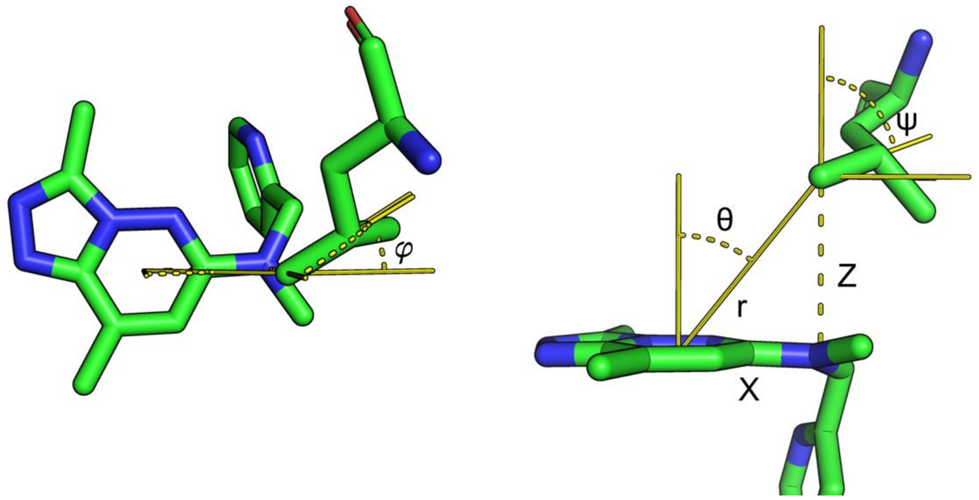

Figure S1: Interaction geometry parameters visualized on Ligand 8. For the pople model only θ and r are used as well as the ring size to select the correct diameter.

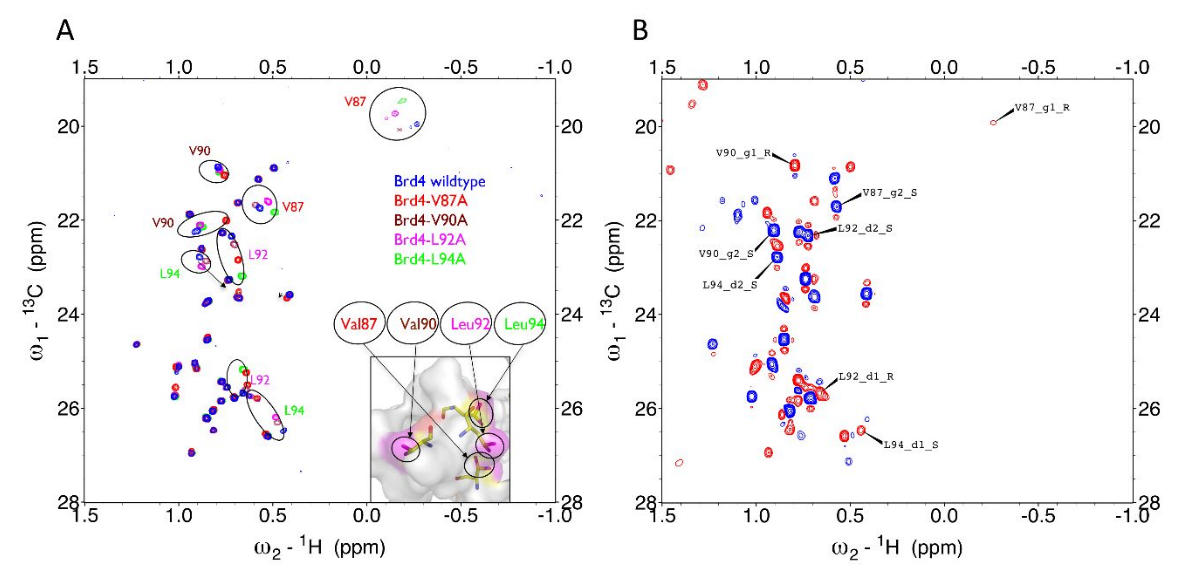

Figure S2: Assignment of selected Leucine and Valine resonances: A: Overlay of the spectrum of wildtype Brd4-BD1 in blue with the spectra of the four Brd4-BD1 mutants (V87A, V90A, L92A, L94A). The structure in (A) shows the 4 residues that are closest to the ligand binding site with V87, L92, L94 facing the ligand. B: Constant time ^{1}H-^{13}C HSQC of fractionally labeled Brd4-BD1 for stereospecific assignment of Leucine Cδ-resonances and Valine Cγ-resonances. Positive resonances (blue) are pro-S methyl groups, negative resonances (red) are pro-R methyl groups of Valine and Leucine residues.

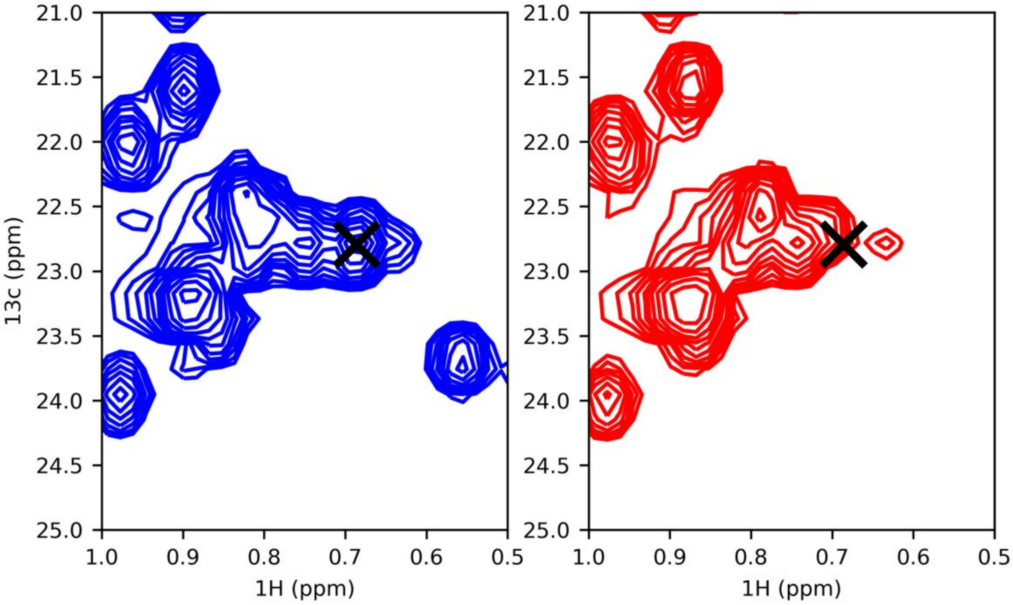

Figure S3: Overlap for position of apo Leu92 $C\delta_1$-$H\delta_1$ resonances (blue) in comparison with ligand 4 bound state (red)

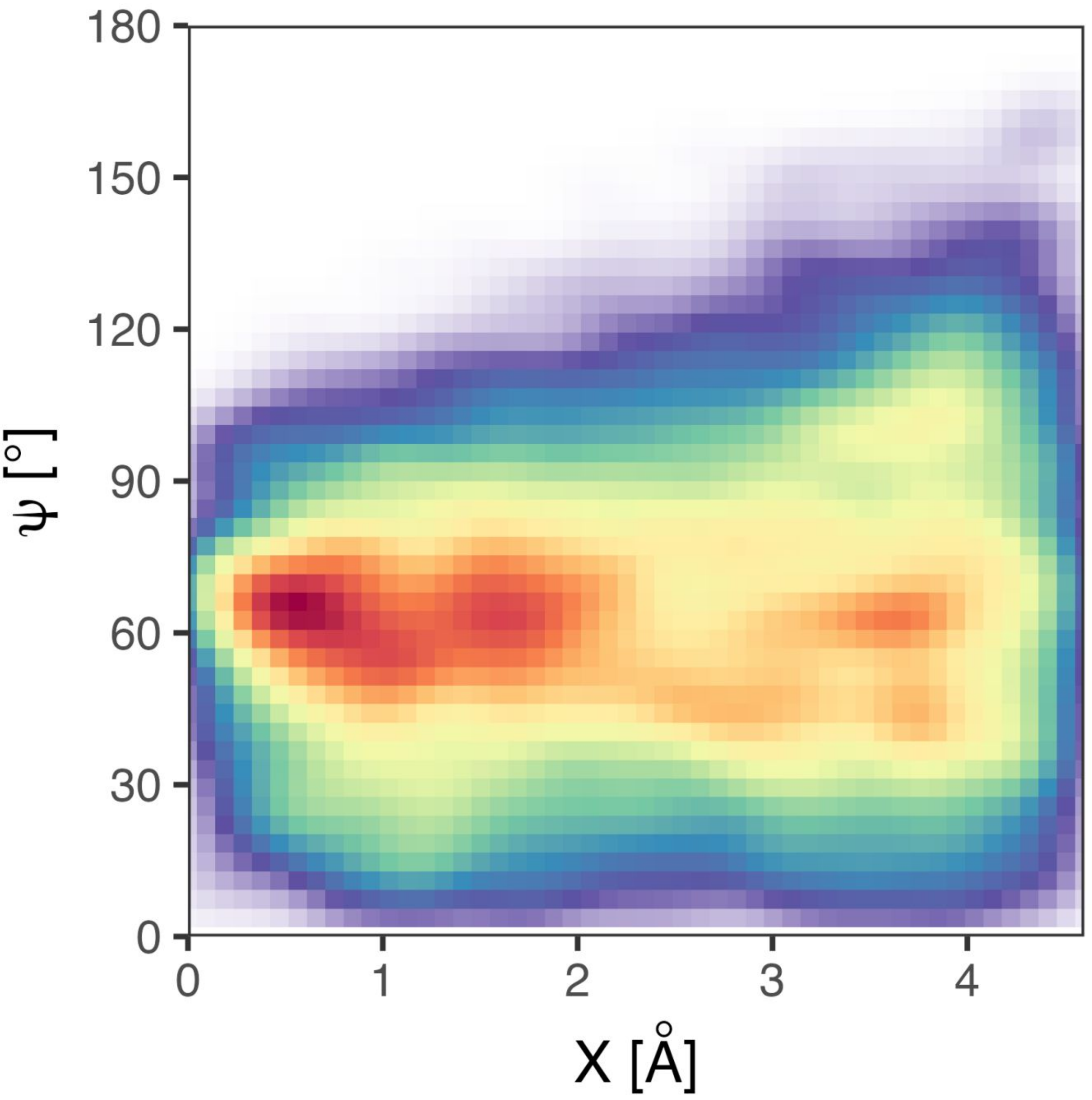

Figure S4: Relative frequency of methyl-vector ψ-values dependent in displacement along the ring plane (X)

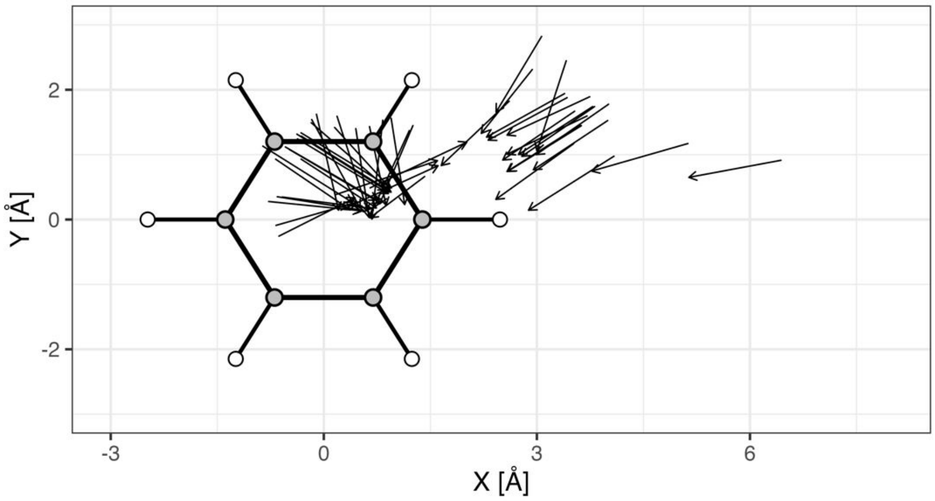

Figure S5: Top-down projection of methyl vectors for ligands 1-10

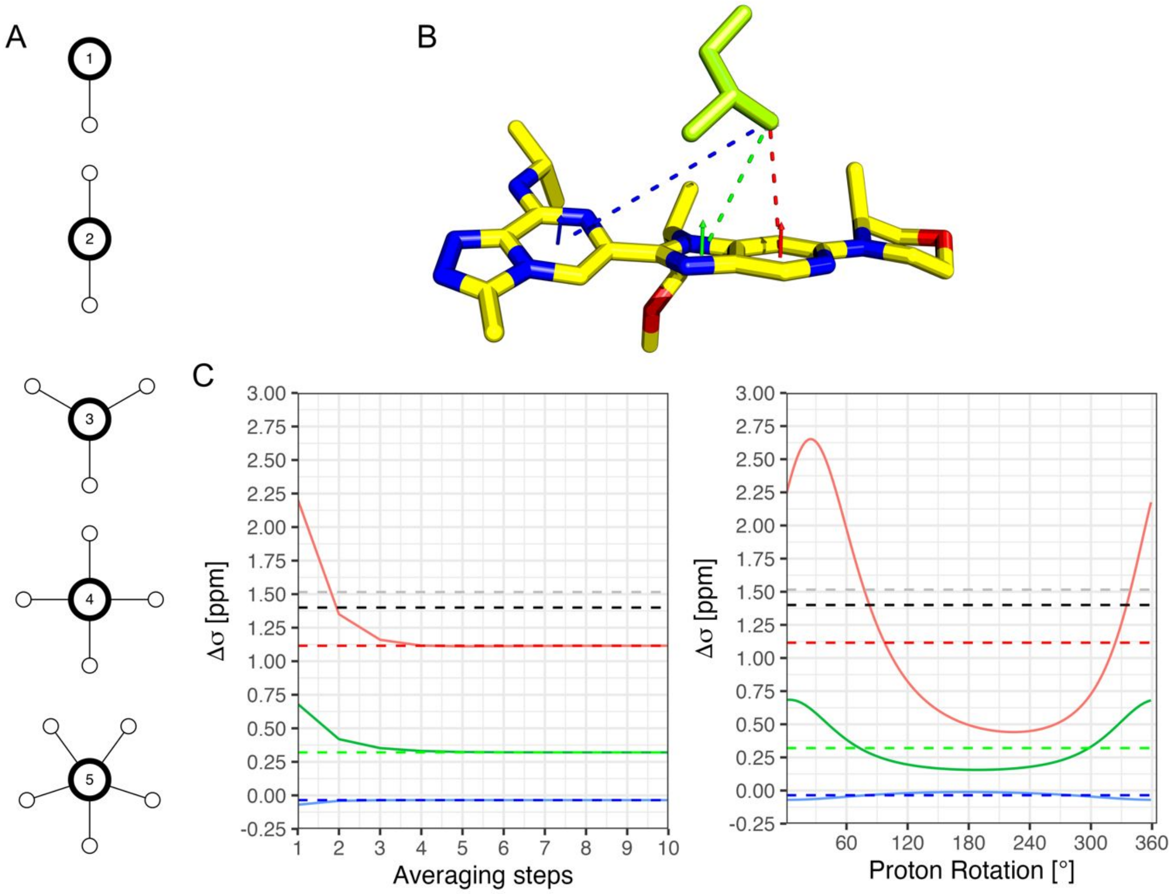

Figure S6: Comparison of calculated shift values based on proton rotation around the C-C symmetry axis. A: For stepwise averaging, the hydrogen is positioned along the normal direction of the ring and consequently rotated by a specified number of equal sized steps around the central C-C symmetry axis. B: For the calculation of the chemical shift change of Leu92-Cδ_2, 3 aromatic rings of ligand 1 within 8 Angstrom are taken into consideration (PDB: 6XUZ). Ring normals and center distances are indicated by arrows and lines. C: Changes in calculated shifts dependent on the number of hydrogen positions averaged over (left, see A) or as a function of the rotation angle for a single hydrogen atom (right). Colored lines represent ring positions as indicated in B. Dashed lines represent the average over 100 uniformly spaced rotation steps. Black and gray lines represent the sum of the averages for all 3 rings and the scaled experimental value respectively.

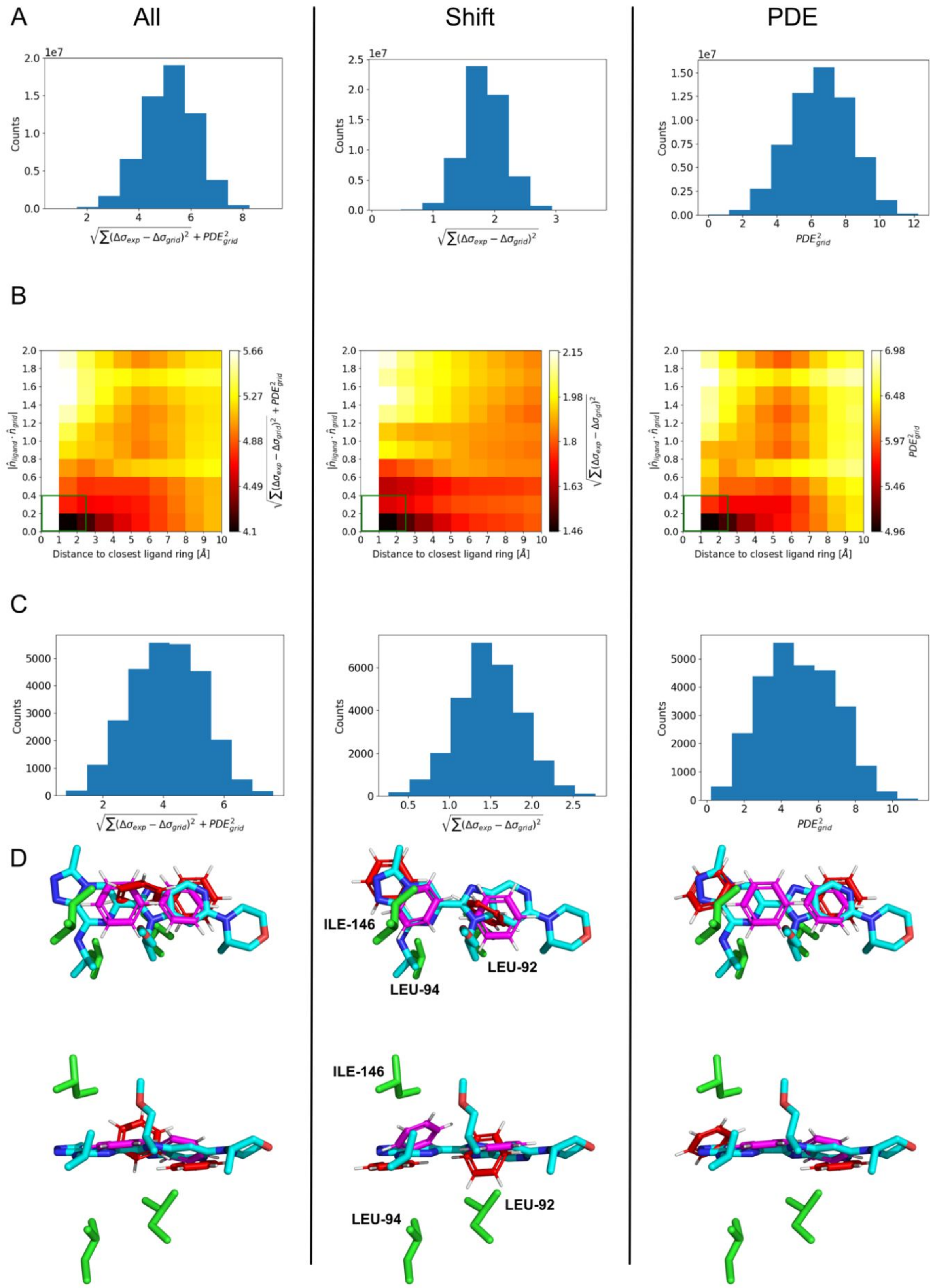

Figure S7: Ring fit statistics for Ligand 1 (PDB: 6XUZ) and the respective shift and PDB probability density estimation components. A: Histogram of the fit score over all grid positions. B: Average fit score binned by the summed distance to the closest ring in the respective crystal structure (x-axis) and the sum of the absolute dot products between the respective ring normals in the fit and the experimental structure (y-axis). C: Histogram of the region indicated by a green box in C. D: Best (magenta) and worst (red) ring position based on fit values in the region indicated by a green box in C. Fitting 2 rings simultaneously and therefore evaluating expanding the single-ring grid by itself leads to a large increase in histogram counts compared to ligands 8 (Figure S8) and 12 (Figure S9)

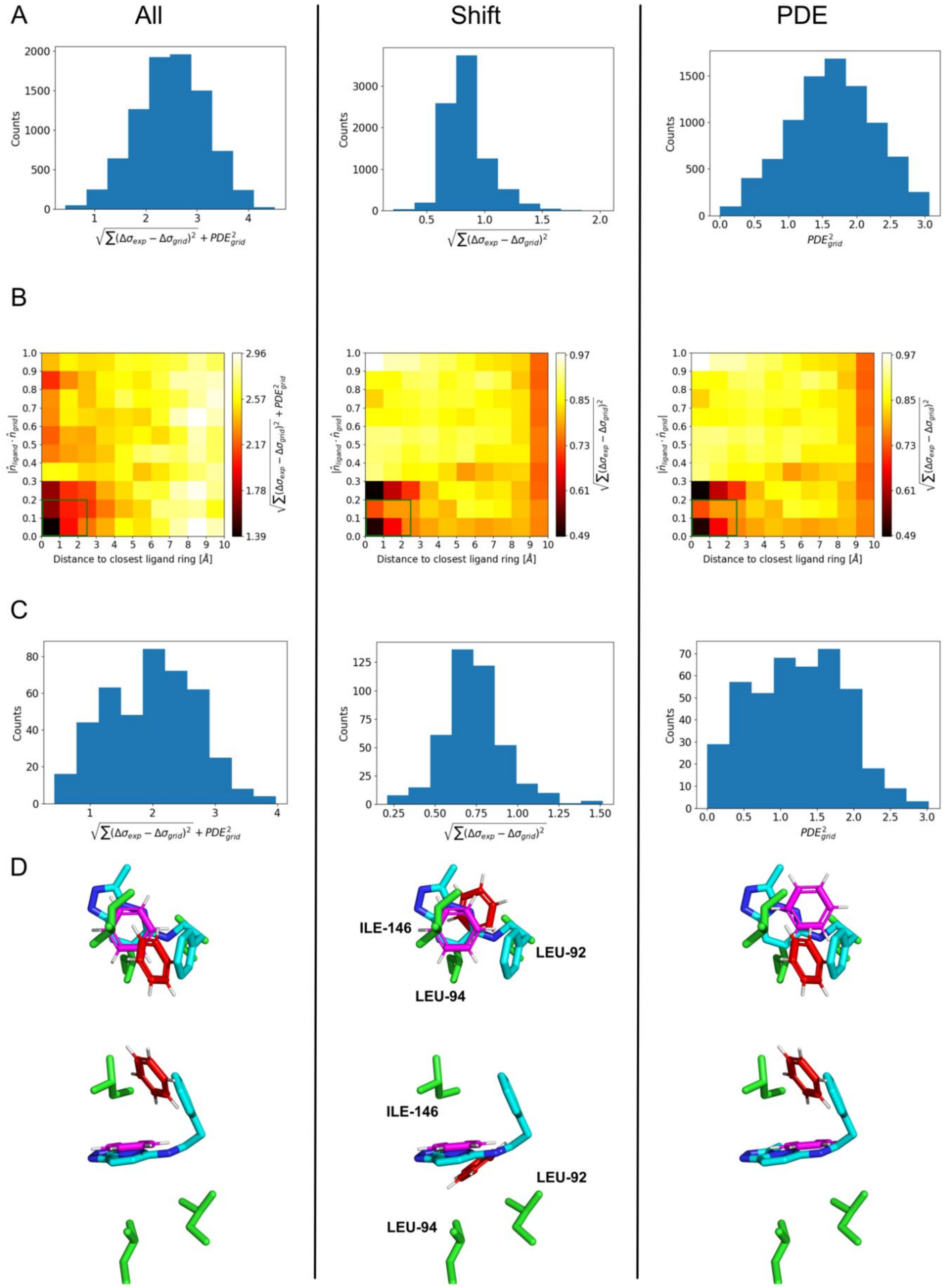

Figure S8: Ring fit statistics for Ligand 8 (PDB: 9FWX) and the respective shift and PDB probability density estimation components. A: Histogram of the fit score over all grid positions. B: Average fit score binned by the distance to the closest ring in the respective crystal structure (x-axis) and the absolute dot products between the respective ring normals in the fit and the experimental structure (y-axis). C: Histogram of the region indicated by a green box in C. D: Best (magenta) and worst (red) ring position based on fit values in the region indicated by a green box in C.

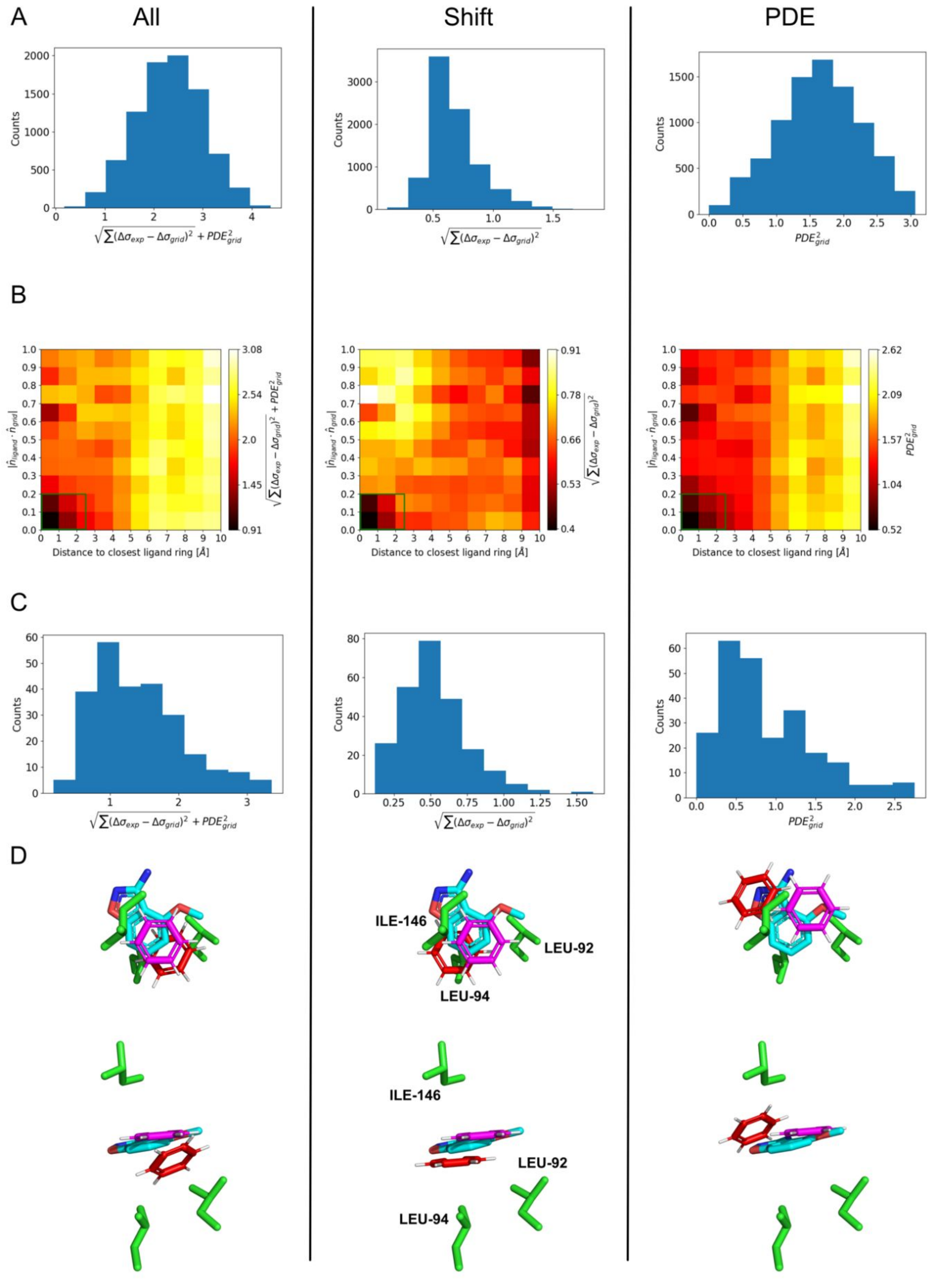

Figure S9: Ring fit statistics for Ligand 12 (PDB: 9FXP) and the respective shift and PDB probability density estimation components. A: Histogram of the fit score over all grid positions. B: Average fit score binned by the distance to the closest ring in the respective crystal structure (x-axis) and the absolute dot products between the respective ring normals in the fit and the experimental structure (y_axis). C: Histogram of the region indicated by a green box in C. D: Best (magenta) and worst (red) ring position based on fit values in the region indicated by a green box in C.

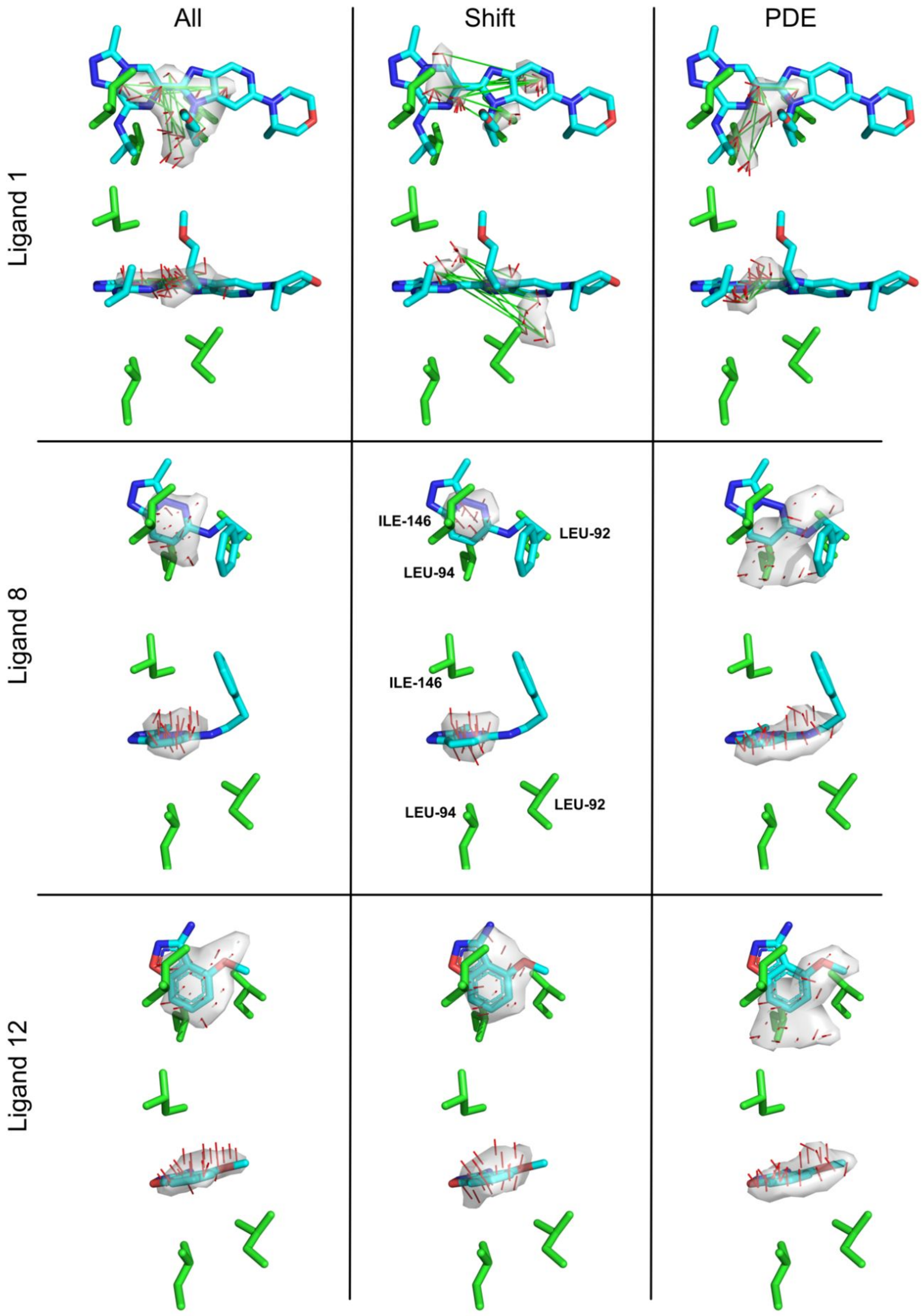

Figure S10: Top and side view of the top 20 result of the ring fit based on overall fit value and its respective shift and PDB probability density estimation components for ligand 1, 8 and 12. Surfaces indicate variability in the ring position while red lines represent the fitted ring normal and therefore ring orientations. For ligand 1, two rings are fit simultaneously, and the green lines indicate connecting vectors between ring pairs.

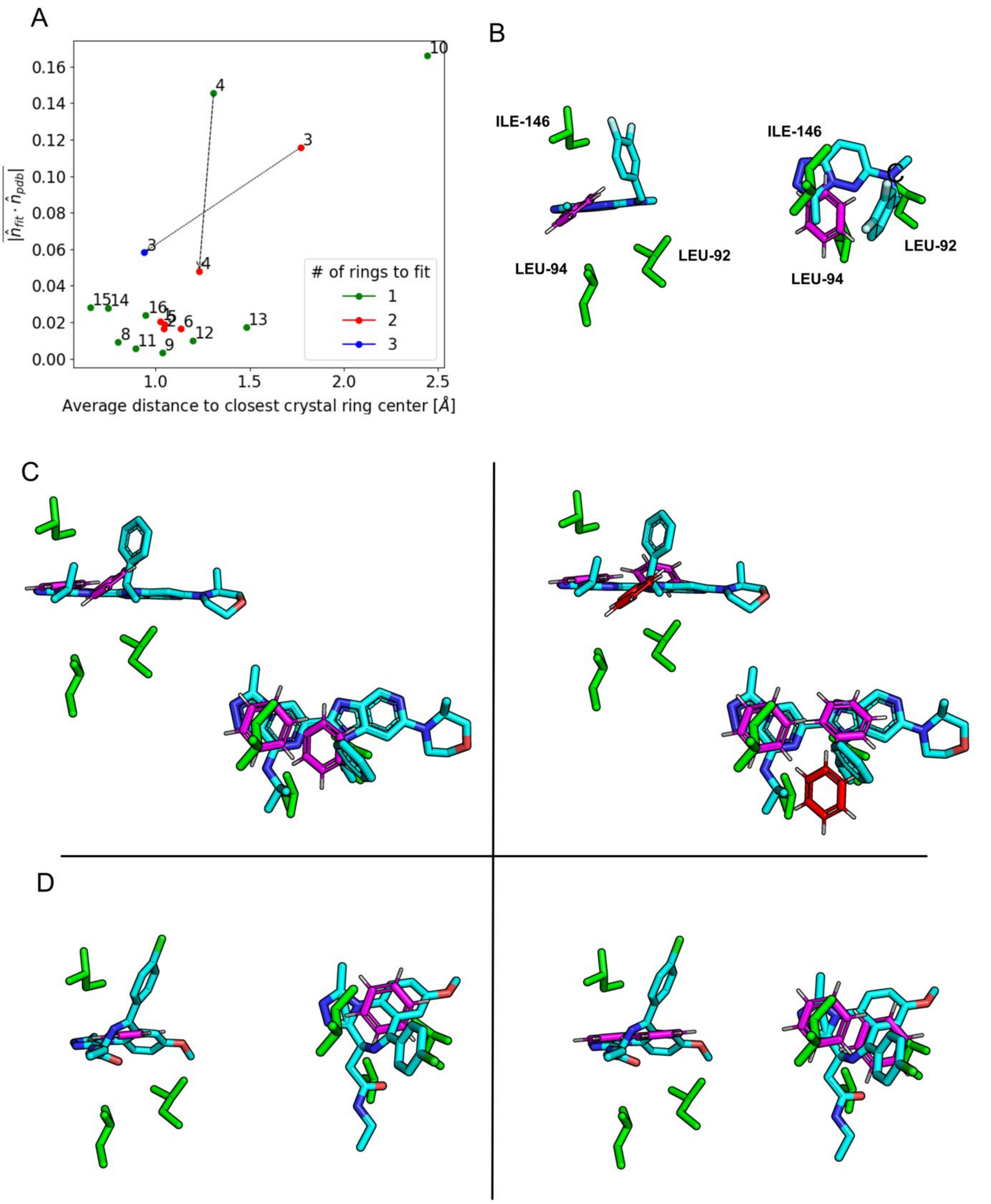

Figure S11: A: Comparison of best ring fit results for the set of ligands. A: Difference in orientation (y-axis) vs distance of ring centers (x-axis) between the best fit and the closest ring in the respective crystal structure. B: For ligand 10 we are missing shift data for Ile-Cδ which leads to a large offset for the best fit. C: For ligand 3, there is an additional ring that has to be accounted for and leads to an intermediate position when fitting only 2 rings (left). When fitting 3 rings (right), the position of the 2 main fit rings improves accordingly, while the 3rd ring covers a potential ring position of the 3rd ligand ring after change of one dihedral angle. D: For ligand 4, the presence of the non-planar 7-membered ring hinders good orientational agreement (left). This is improved by including a second ring in the fit (right).

HPLC chromatograms for compounds tested:

All samples were analyzed on an Agilent 1200 series LC system coupled with an Agilent 6140 mass spectrometer. Purity was determined via UV detection with a bandwidth of 170nm in the range from 230-400nm. LC parameters were as follows: Waters Xbridge C18 column, 2.5µm particle size, 2.1 x 20mm. Run time 2.1 minutes, flow 1ml/min, column temperature 60°C and 5µl injections. Solvent A (20mM NH4HCO3/ NH3 pH 9), solvent B (MS grade acetonitrile). Start 10% B, gradient 10% - 95% B from 0.0 - 1.5min, 95% B from 1.5 - 2.0min, gradient 95% - 10% B from 2.0 – 2.1min.

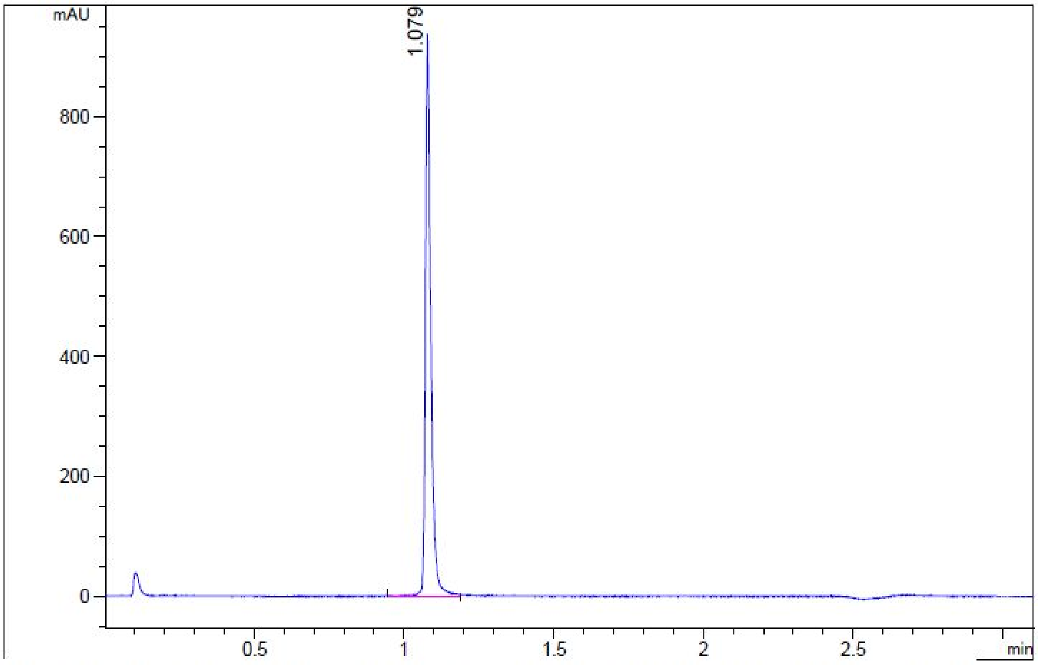

RT: 1.08 min   Area %:   100.0 %

Figure S12: HPLC trace of Ligand 1

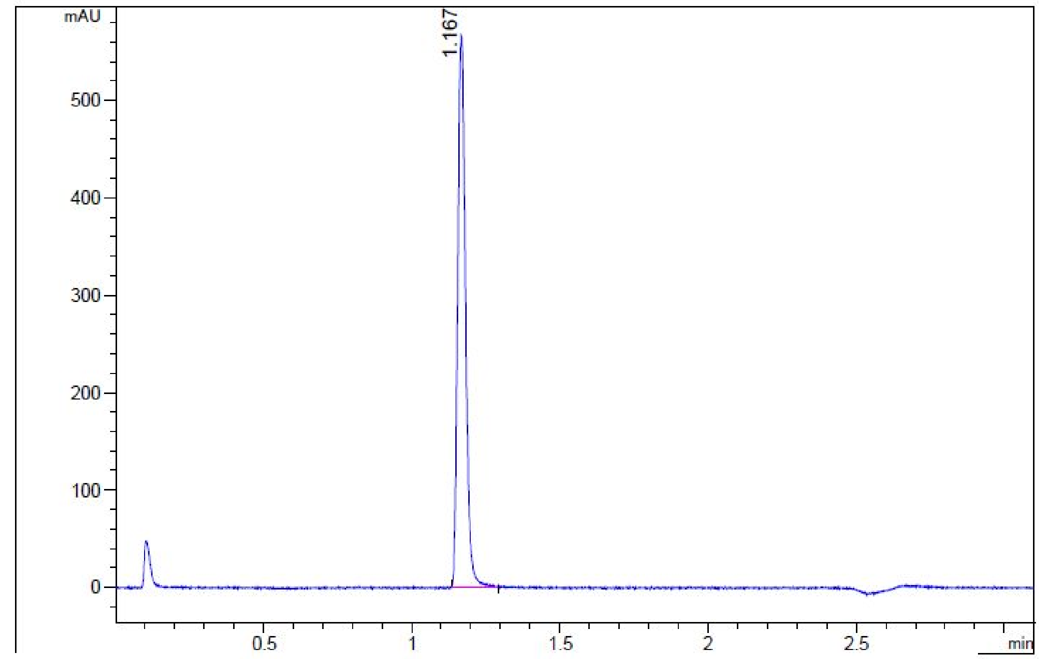

RT: 1.17 min Area %: 100.0 %

Figure S13: HPLC trace of Ligand 2

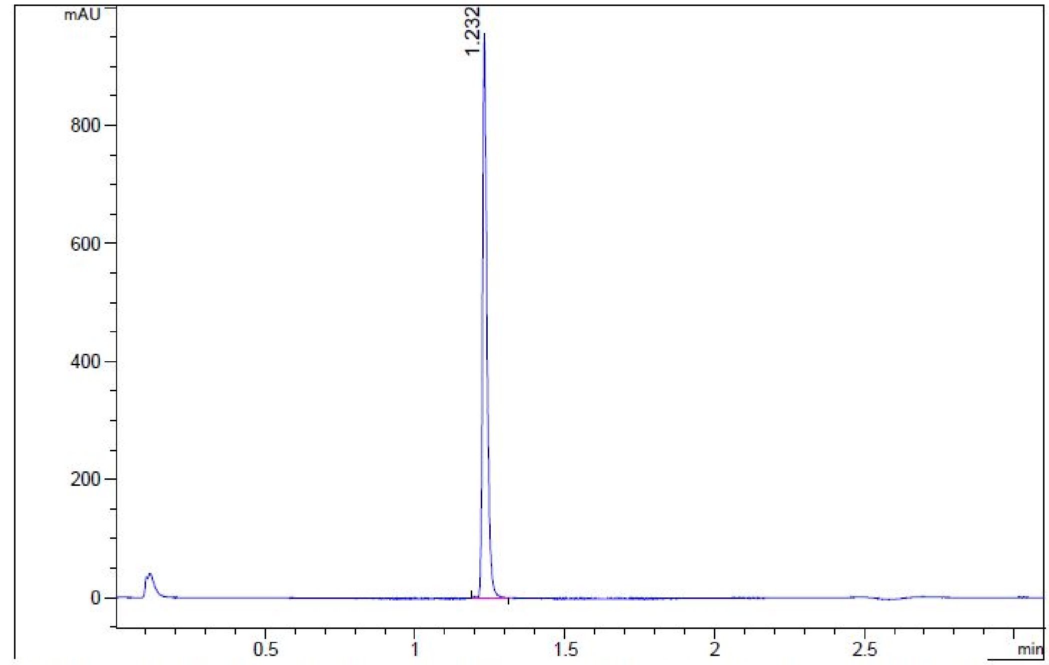

RT: 1.23 min Area %: 100.0 %

Figure S14: HPLC trace of Ligand 3

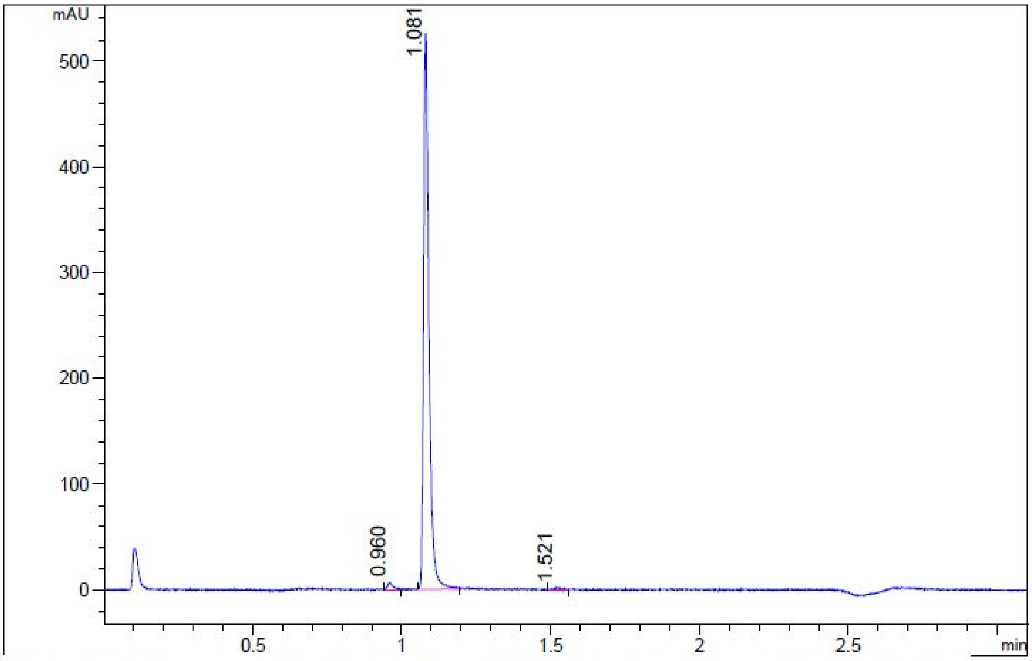

RT: 0.96 min Area %: 1.2 %
RT: 1.08 min Area %: 98.4 %
RT: 1.52 min Area %: 0.4 %

Figure S15: HPLC trace of Ligand 4

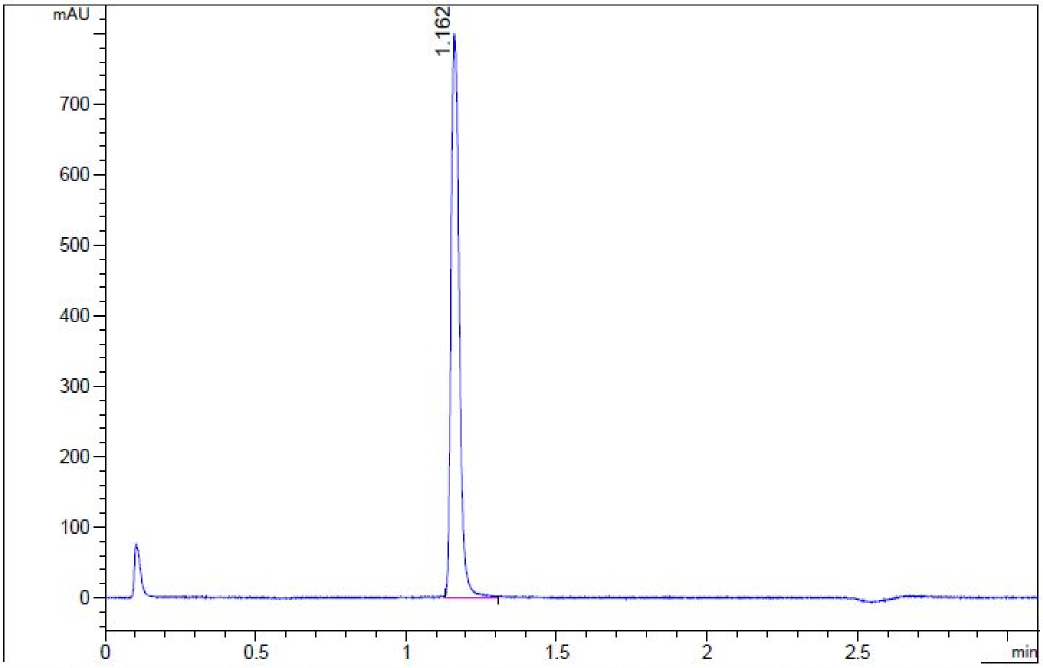

RT: 1.16 min Area %: 100.0 %

Figure S16: HPLC trace of Ligand 5

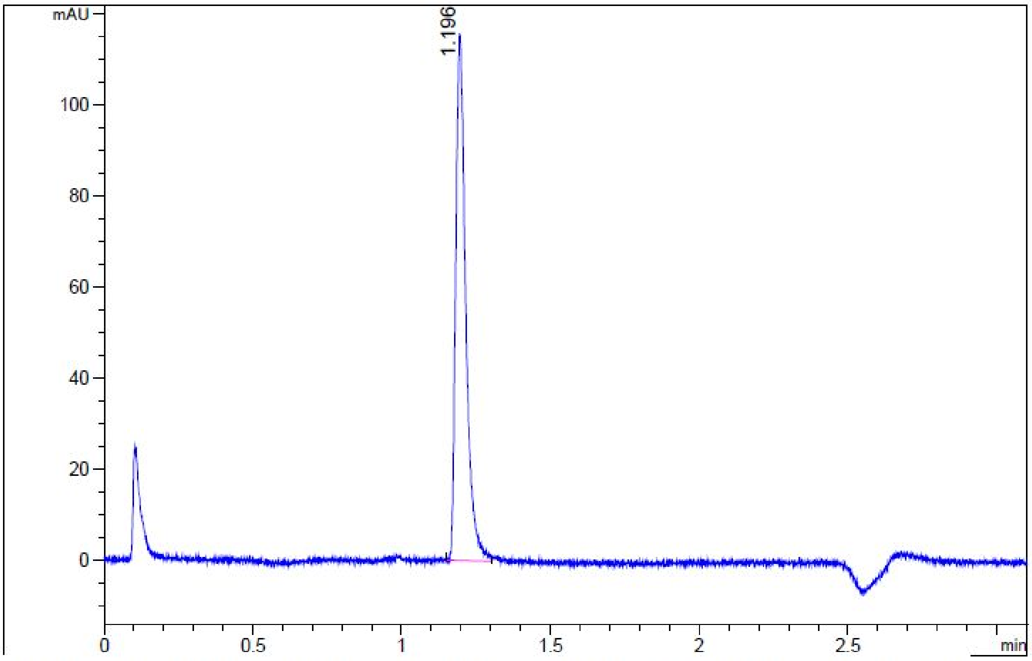


```
----------------------------------------------------------------------------
RT:   1.20 min                          Area %:    100.0 %
----------------------------------------------------------------------------
```

Figure S17: HPLC trace of Ligand 6

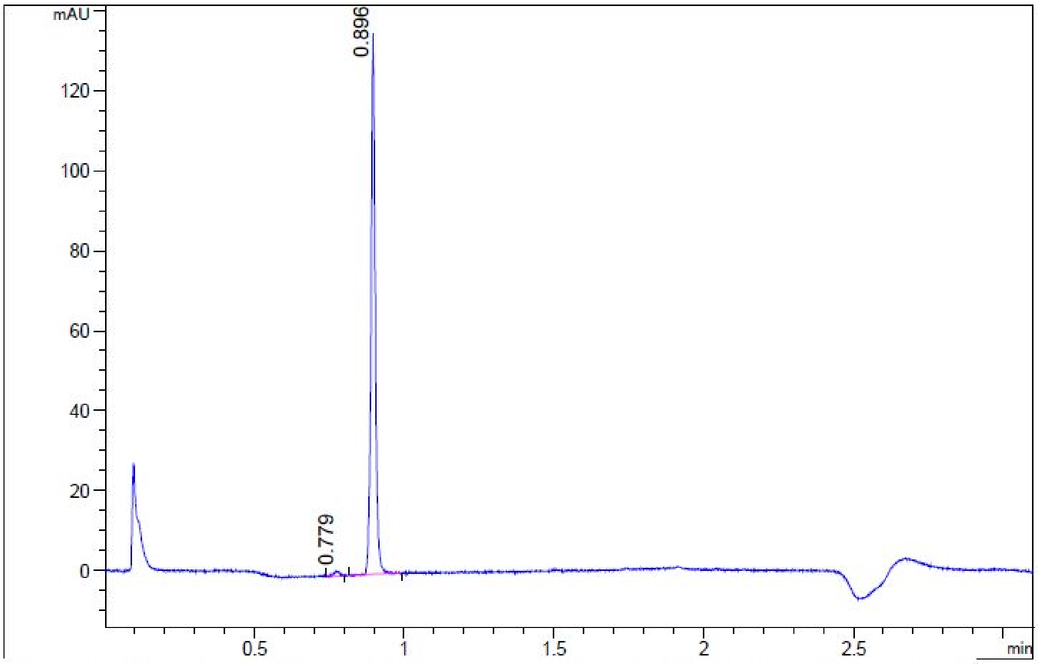


```
----------------------------------------------------------------------------
RT:   0.78 min                          Area %:      1.5 %
RT:   0.90 min                          Area %:     98.5 %
----------------------------------------------------------------------------
```

Figure S18: HPLC trace of ligand 7

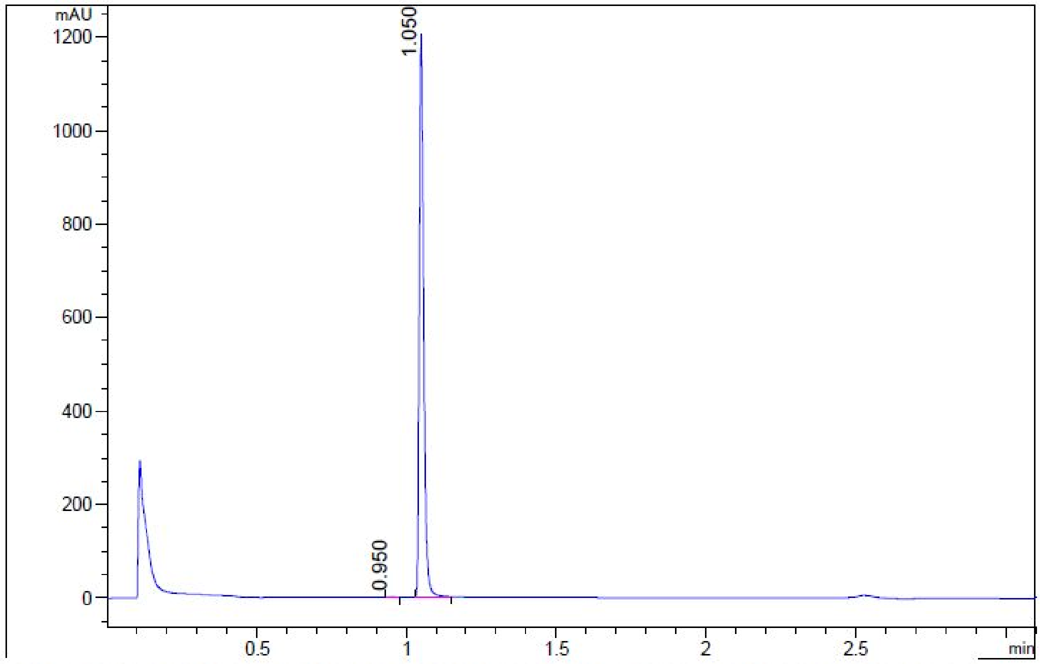

RT:	0.95 min	Area %:	0.1 %
RT:	1.05 min	Area %:	99.9 %

Figure S19: HPLC trace of ligand 8

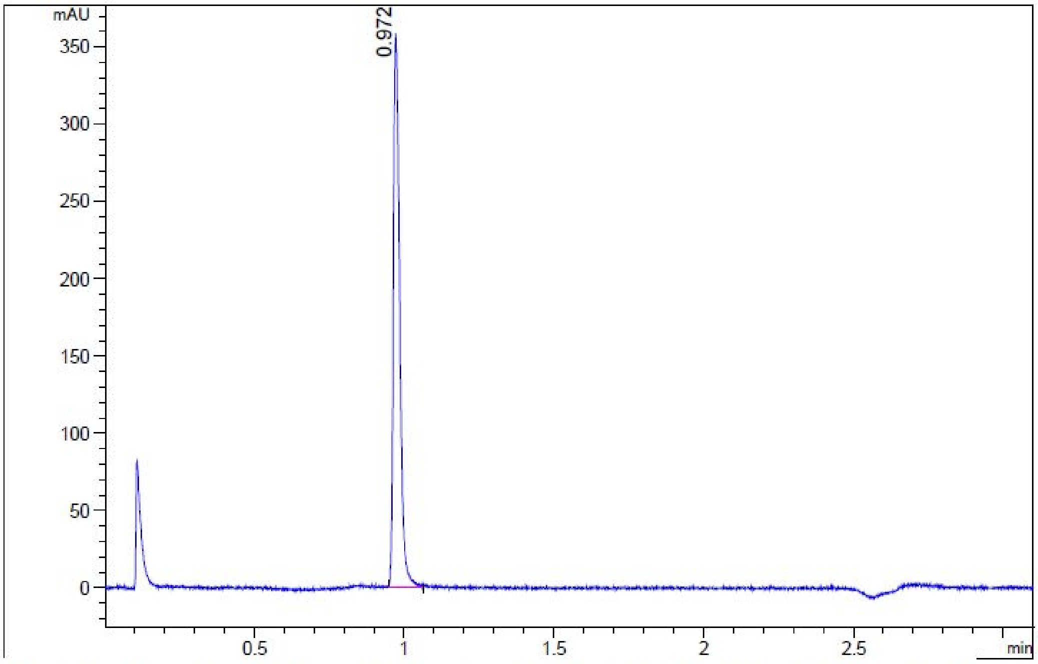

RT:	0.97 min	Area %:	100.0 %

Figure S20: HPLC trace of ligand 9

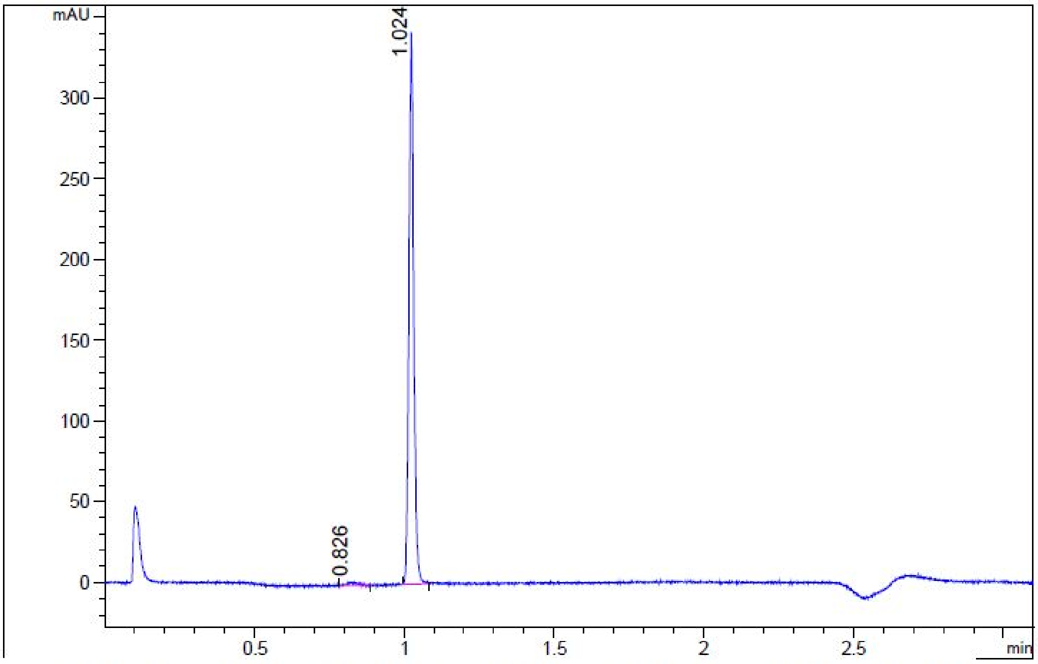


```
RT:   0.83 min                         Area %:     0.9 %
RT:   1.02 min                         Area %:    99.1 %
```

Figure S21: HPLC trace of ligand 10
